# Redox-Divergent Reactivity of a Gallium(I) Ambiphile toward an Allene, a Diyne, and Ph_3_PCN_2_

**DOI:** 10.1021/acs.inorgchem.6c03200

**Published:** 2026-08-10

**Authors:** Simon H. F. Schreiner, Jan N. Ludwig, Tobias Rüffer, Max M. Hansmann, Robert Kretschmer

**Affiliations:** † Institut für Chemie, 38869 Technische Universität Chemnitz, Strasse der Nationen 62, Chemnitz 09111, Germany; ‡ 14311 Technische Universität Dortmund, Otto-Hahn-Straße 6, Dortmund 44227, Germany; § Jena Center of Soft Matter, Friedrich-Schiller-Universität Jena, Philosophenweg 7, Jena 07443, Germany

## Abstract

Main-group element compounds featuring a low-valent metal center typically undergo oxidative addition reactions with organic substrates, while processes without a change of the oxidation state remain rare. Here, we present a study of the ability of our recently reported gallanediyl **1** to undergo carbometalation reactions with an allene and a diyne. In either case, selective 1,2-*syn*-addition reactions with retention of the low oxidation state and the singly bonded nature of the gallium center are observed. In contrast, with the diazophosphorus ylide Ph_3_PCN_2_ an oxidative cycloaddition reaction takes place and gives rise to the C, Ga, N, P-heterocycle **4**. These advances hint toward the utilization of low-valent main-group element compounds in catalytic transformations that are hitherto limited to noble metals.

## Graphical abstract

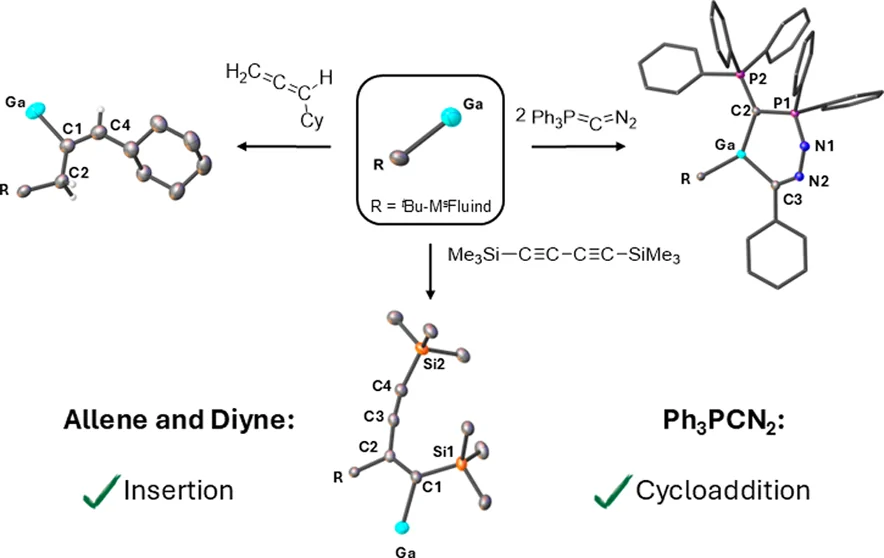

## Introduction

Aiming to mimic the behavior of transition metals, research on low-valent compounds of the main-group elements has progressed significantly over the past decades.^1^ While catalytic transformations featuring two-electron redox processes such as oxidative addition and reductive elimination have been considered as the exclusive playground of noble metals, recent results featuring Group 15 elements,^2^ highlight the hitherto hidden potential of main-group elements in redox catalysis. However, unfavorable thermodynamics promote unidirectional reactions and catalytic transformations traversing through sequences of reductive elimination and oxidative addition reaction remain challenging.^3^ In addition to these two-electron shuttling processes, the redox-invariant *syn*-1,2-insertion reaction, Figure 1a^4^ also constitutes a crucial part of many cycles in noble-metal catalysis.^5^ When it comes to main-group elements carbometalation reactions, i.e., the insertion of unsaturated substrates into a main-group element–carbon bond remain rare,^6^ although they constitute the core of Ziegler’s Aufbau reaction, Figure 1b.^7^ In contrast, the 1,2-insertion into main-group element–heteroatom bonds is more frequently observed and plays a prominent role in hydrofunctionalization reactions for example of s-block element compounds.^8^ When joined with σ-bond metathesis, it even enables redox-invariant catalytic transformations.^9^ In contrast to the broad utilization of 1,2-insertions of compounds with main-group elements in high oxidation states, reports on carbometalation reactions of low-valent main-group element compounds remain very rare and are limited to Group 14 and 15 elements.^10^ In case of the Group 13 elements, however, oxidative addition is typically the predominant reaction pathway^3b,11^ and until our recent report on the reactivity of a gallanediyl with alkynes, Figure 1c,^10e^ redox-invariant reactions remained limited to the formation of Lewis acid–base pairs.^12^ Very recently, the Goicoechea group reported on a 1,2-insertion of a phosphaalkyne into an In–C bond.^13^ We hypothesized that the carbometalation may not be limited to alkynes and considered diynes as well as allenes as interesting substrates given that their insertion is one of the most important elementary steps in many catalytic reactions and also because the insertion of allenes into transition-metal–carbon bonds often gives rise to π-allyl complexes.^14^ Furthermore, application of the recently discovered diazophosphorus ylide Ph_3_PCN_2_ ^15^ could grant access to interesting new reactivity. Extending the concept of carbometalation by low-valent main-group element compounds would leverage current constraints and help to design new catalytic reactions in the future.

**Figure 1:**
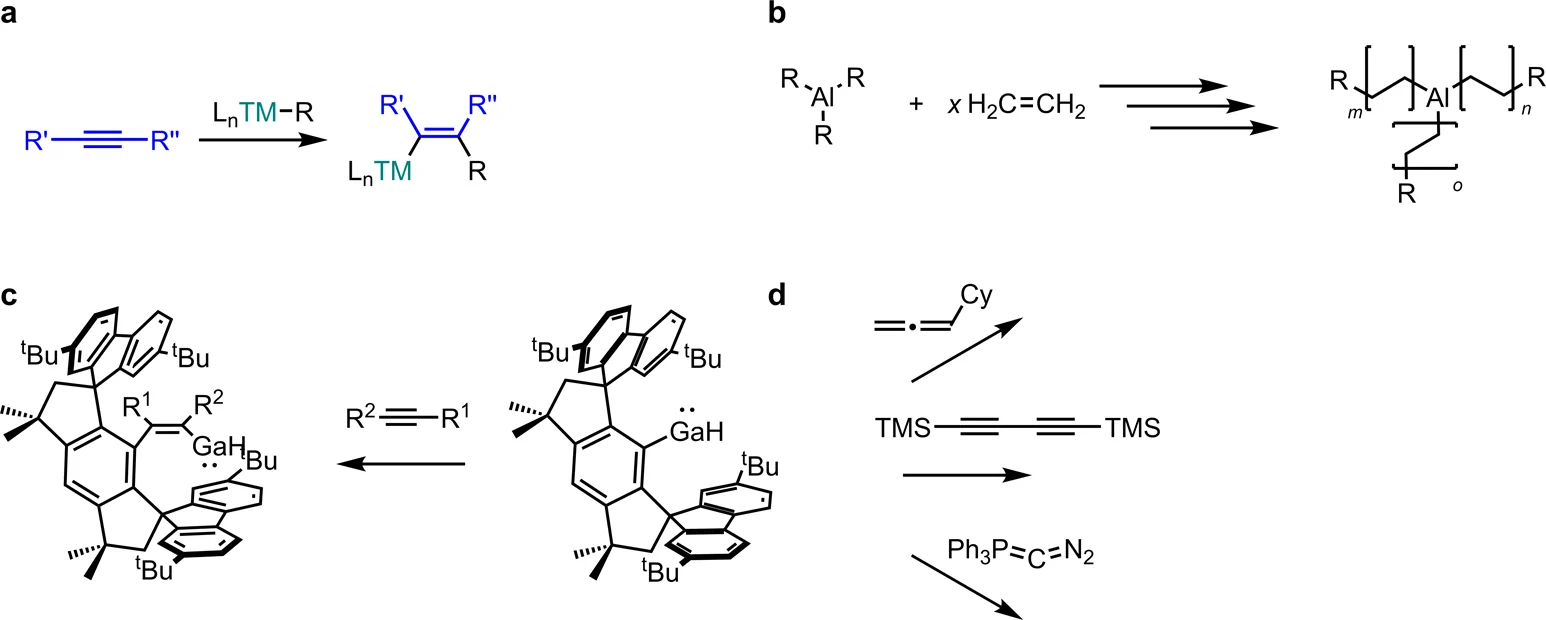
Examples of carbometalation reactions of a) transition-metal complexes and b) aluminum­(III) compounds. c) 1,2-insertion of alkynes into the Ga–C bond of a gallanediyl and d) its reactivity toward an allene, diyne, and Ph_3_PCN_2_ studied in here.

## Results and Discussion

The freshly prepared gallanediyl **1** ^10e^ readily reacts with one equivalent cyclohexylallene at 50 °C, Scheme 1, and the conversion is completed after 16 h according to the ^1^H NMR spectrum. The yellow solution was filtered in order to remove remaining particles and slow evaporation afforded yellow crystals that were washed with *n*-pentane to derive **2** in 54% yield. Notably, a minor fraction of the proteo-ligand ^*t*^Bu-M^s^Fluind–H is also present and could not be removed despite repeated attempts; a similar behavior has previously been reported by Beckmann and coworkers for hydrindacene zinc complexes.^16^ The ^1^H NMR spectrum features one set of resonances in account for the selective formation of one compound and the resonance pattern agrees well with a carbometalation of the terminal double bond. In detail, a doublet at 1.99 ppm with a coupling constant of *J* = 2.8 Hz and a doublet of triplets (*J* = 7.2 and 2.7 Hz) at 3.82 ppm account for the newly formed allyl group. The molecular structure in the solid state, Figure 2a, was established by X-ray diffraction (XRD) analysis and agrees well with the observations by ^1^H NMR spectroscopy. The reaction proceeds with high selectivity affording exclusively the 2,3-*syn*-addition product **2**. This is a remarkable difference to the behavior of transition metals as they typically afford π-allyl complexes,^14^ which is due to the availability of suitable empty d-orbitals at transition metal centers that can interact with the bonding and nonbonding π-orbitals of the allyl. In case of gallium, the 3d-orbitals are fully occupied and only an interaction with the ally π*-orbital might be considerable. The Ga–C bond length of 2.007(3) Å is slightly shorter as in the carbometalation products of **1** and alkynes (2.042(3)–2.0711(19) Å)^10e^ but still slightly exceeds those of vinylic gallium­(III) compounds.^17^ The closest distance of the gallium center to a carbon atom of the flanking fluorenyl rest, i.e., Ga···C43, amounts to 3.159(3) Å, which indicates dispersive stabilization as observed previously.^10e^

**Scheme 1:**
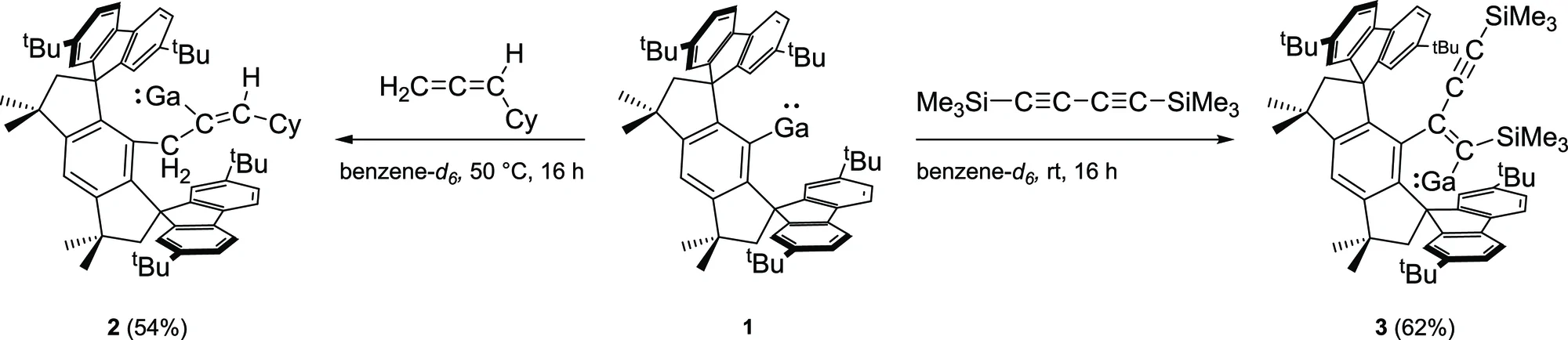
Reactions of the Gallanediyl **1** with Cyclohexylallene and 1,4-Bis­(trimethylsilyl)-1,3-butadiyne Afford the Carbometallation Products **2** and **3**, Respectively

**Figure 2:**
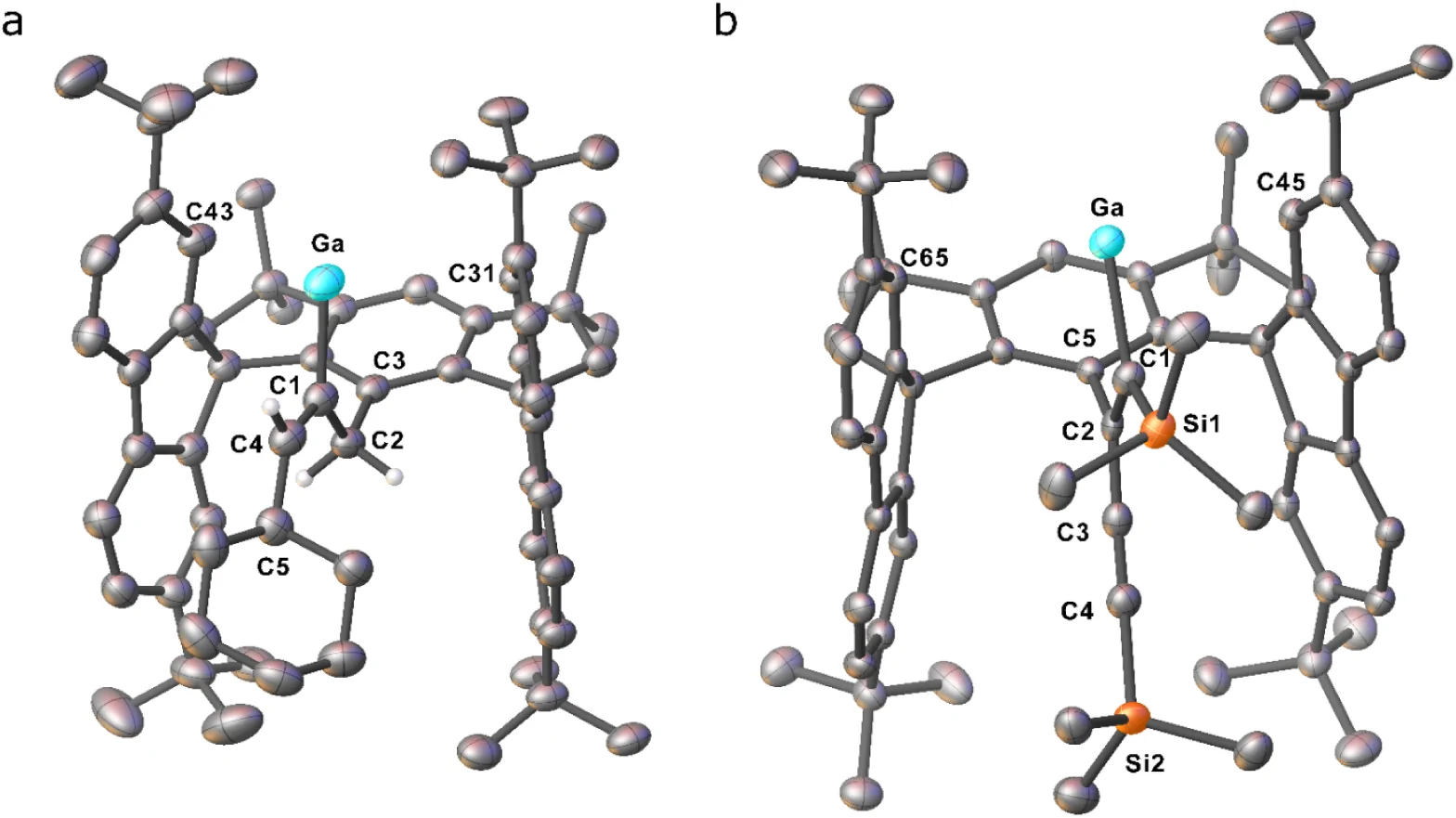
Solid-state molecular structures of **2** (a) and **3** (b) (ellipsoids represent 50% probability). Selected bond lengths/distances (Å) of **2** (a): C1–Ga 2.007(3), C1–C2 1.497(4), C2–C3 1.533(4), C1–C4 1.345(5), C4–C5 1.505(5), Ga···Ga 9.735, Ga···C43 3.159(3), Ga···C31 3.224(3). A second molecule of **2** in the asymmetric unit (with *^t^* Bu and cyclohexyl disorder) and hydrogen atoms are omitted for clarity. Selected bond lengths/distances (Å) and angles (°) of **3** (b): C1–Ga 2.072(2), C1–C2 1.359(3), C2–C3 1.423(3), C3–C4 1.215(3), C2–C5 1.543(2), C1–Si1 1.8762(19), C4–Si2 1.833(2), C2–C3–C4 171.8(2), C3–C4–Si2 167.77(19), Ga···Ga 9.255, Ga···C45 2.9553(18), Ga···C65 3.2005(18). Second molecule of **3** in asymmetric unit with disordered *^t^* Bu group, two benzene solvent molecules and hydrogen atoms are omitted for clarity.

Next, we allowed **1** ^10e^ to react with one equivalent of 1,4-bis­(trimethylsilyl)-1,3-butadiyne. Similar to the reaction with alkynes,^10e^ conversion is already achieved at room temperature and completed after 16 h. Workup similar to **2** afforded **3** in 62% yield. The ^1^H, ^13^C and ^29^Si NMR spectra hint toward a monocarbometalation product. This is illustrated by two singlets in both the ^1^H (δ = 0.05 and −0.25 ppm) and the ^29^Si DEPT-135 (δ = −13.6 and −21.1 ppm) NMR spectra, which account for two chemically and magnetically inequivalent trimethylsilyl groups. The presence of an intact alkyne group is substantiated by the respective resonances in the ^13^C­{^1^H} NMR spectrum at δ = 94.9 and 105.2 ppm, respectively. Single-crystals suitable for an XRD analysis were obtained directly from the filtered reaction solution upon evaporation of the solvent; the molecular structure in the solid state is depicted in Figure 2b. As indicated by NMR spectroscopy, only one of the two alkyne groups reacted. The reaction affords the *syn*-addition product and proceeds with high regioselectivity, affording exclusively **3**, in which gallium is bound to the α-carbon atom with respect to the trimethylsilyl group. This observation aligns well with the α-silyl effect and a better stabilization of the partial negative charge. The Ga–C bond length of 2.072(2) Å is reminiscent of those previously reported for the carbometalation products of **1** and alkynes (2.042(3)–2.0711(19) Å).^10e^ Within **3**, even shorter Ga···C contacts with the adjacent fluorenyl wings are recognized, with Ga···C45 being the shortest (2.9553(18) Å). Finally, we attempted to enforce a second carbometalation but using the gallanediyl **1** in excess and under more forcing conditions only gives rise to mixtures of **3** and unreacted **1**. This is likely caused by the steric protection of the alkyne by the flanking fluorenyl rests, Figure 2b, which hinders an interaction with a second gallium­(I) center.

Finally, we investigated the reactivity of **1** toward the diazophosphorus ylide Ph_3_PCN_2_ ^15^ previously reported by the Hansmann group with the aim to establish a ^*t*^Bu-M^s^FluindGa=CPPh_3_ bond upon cleavage of dinitrogen. The reaction of **1** and Ph_3_PCN_2_ proceeds already at room temperature, and gas bubbles indicate the formation of dinitrogen. With a 1:1 stoichiometry, the formation of two compounds is recognized based on the ^31^P­{^1^H} NMR spectrum, which features two sets of resonances, i.e., a singlet at −10.3 ppm as well as two doublets (^2^ *J*_*P,P*_ = 53.7 Hz) at 6.1 and −6.0 ppm, respectively, in a ratio of about 0.26:1:1. In order to gain some insight into the structural relationship between both compounds, we used ^31^P diffusion-ordered NMR spectroscopy (DOSY), Figure S3. The DOSY signals in the current case are broad but nevertheless well-resolved. The diffusion coefficient of 4.14 × 10^–10^ m^2^ s^–1^ assigned to the singlet resonance at −10.3 ppm is only slightly larger than those of the two doublets, i.e., 3.60 × 10^–10^ m^2^ s^–1^ to 3.95 × 10^–10^ m^2^ s^–1^. Following the approach established by Stalke and co-worker^18^ as well as Hiller et al.^19^, they account for a molar mass of about 1190 g mol^–1^ and 1181 g mol^–1^, respectively, as well as 1283 to 1490 g mol^–1^ and 1273 to 1468 g mol^–1^, respectively, when using the parameter for dissipated spheres and ellipsoids; see the Supporting Information for details. Hence, the singlet could be assigned to a 1:1 adduct of the Lewis-basic Ph_3_PCN_2_ and the Lewis amphoteric **1**. In contrast, the compound causing the two doublets is likely formed from the reaction of **1** with two molecules of the diazophosphorus ylide, which is further substantiated by the observation of phosphorus–phosphorus coupling. To gain insight into the interplay of the two compounds, low-temperature ^31^P­{^1^H} NMR experiments have been conducted, Figure S4. Formation of both compounds was already recognized at −75 °C and full consumption of Ph_3_PCN_2_ was achieved at −45 °C. Hence, the selective formation and isolation of a formal 1:1 adduct seems not possible by kinetic control. Furthermore, low-temperature *in situ* IR spectroscopy was conducted, Figure S5. The distinct vibration of Ph_3_PCN_2_ can be recognized at 1995 cm^–1^ and decays over time. However, no indication of a second compound featuring a N=N double bond is observed in the spectra. This could argue against a C-bonded 1:1 adduct ^*t*^Bu-M^s^FluindGa–C­(N_2_)­PPh_3_ but given the low concentration it might be hidden below other bands.

Aiming to enforce the formation of a single product and to substantiate the claim that the product is formed from a 1:2 stoichiometry, the reaction was repeated with an initial 1:2 ratio of **1** and Ph_3_PCN_2_, Scheme 2. Now, full conversion was observed within 16 h forming exclusively one product. This features the same ^1^H and ^31^P­{^1^H} NMR resonances as observed in the spectra of the 1:1 reaction, and accounts for the compound with the higher molecular mass. After filtration of the reaction mixture, orange single-crystals of **4** could be grown by slow evaporation and **4** was obtained in overall 64% yield. Its molecular structure in the solid state was determined by XRD analysis and is shown in Figure 3. **4** features an unprecedented C, Ga, N, P-heterocycle which is formally constructed from **1** and two molecules of Ph_3_PCN_2_ through concomitant loss of one molecule of dinitrogen and migration of both a PPh_2_ and a Ph fragment. **4** is best described by the two resonance forms shown in Scheme 2, featuring phosphonium ylides. Related P–C–P phosphonium ions have been reported before.^20^

**Scheme 2:**
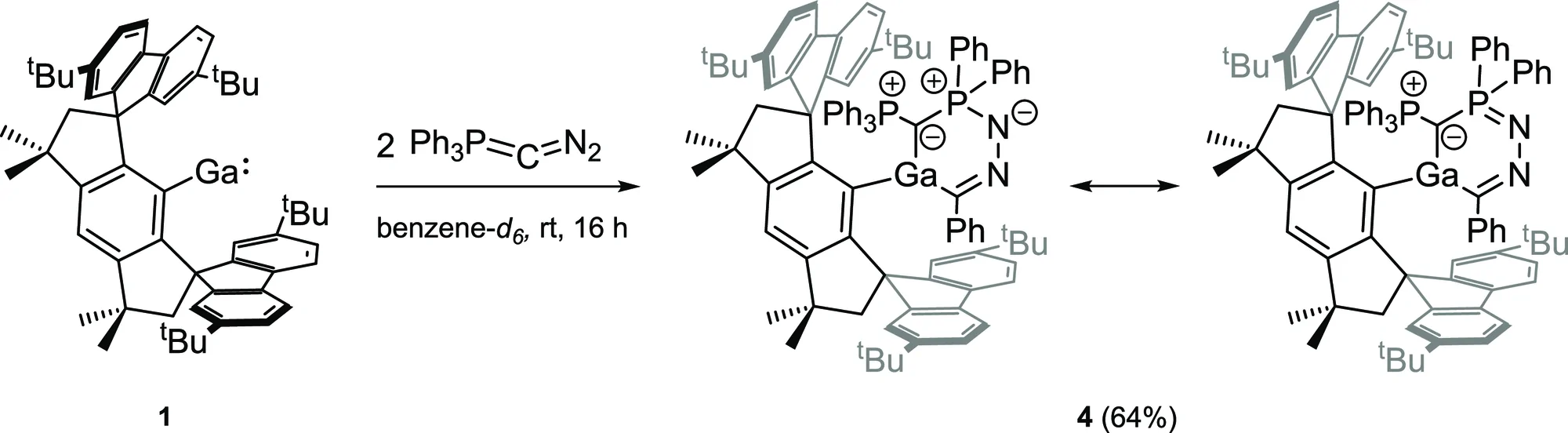
Reaction of the Gallanediyl **1** with the Diazophosphorus Ylide Ph_3_PCN_2_ Gives Rise to the Heterocycle **4**

**Figure 3:**
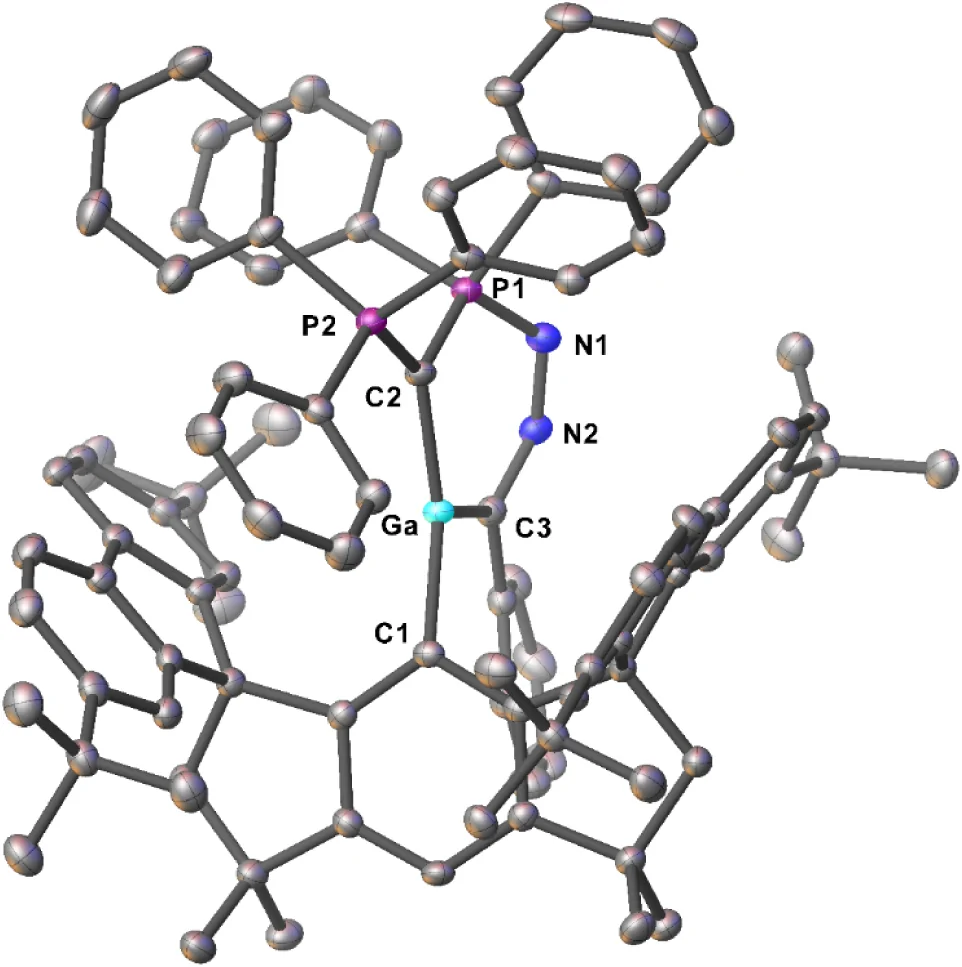
Solid-state molecular structure of **4** (ellipsoids represent 50% probability). Selected bond lengths (Å) and angles (°): C1–Ga 1.9988(18), Ga–C2 1.9278(18), Ga–C3 1.9911(19), C2–P1 1.7743(18), C2–P2 1.7204(19), P1–N1 1.6206(16), N1–N2 1.352(2), N2–C3 1.313(2), Ga–C2–P1 107.10(9), P2–C2–P1 118.98(10), P2–C2–Ga 133.92(10), C2–P1–N1 117.75(8), P1–N1–N2 127.87(13), N1–N2–C3 124.93(16), N2–C3–Ga 120.84(14), C2–Ga–C3 110.54(8). Hydrogen atoms are omitted for clarity.

The endo- (C2–P1 1.7743(18) Å) and exocyclic (C2–P1 1.7743(18) Å) carbon–phosphorus bonds fall in the range reported for a phosphonium salt of the type [(PPh_3_)_2_CPPh_2_]^+^ (1.74(1) to 1.811(1) Å) reported before.^20d^ However, they are longer compared to those of neutral (1.6951(17) to 1.7355(17) Å) and metalated (1.627(2) to 1.656(2) Å) acyclic ylides.^20e^ The observation that C2–P1 is significantly longer than C2–P2 reflects previous reports on the phosphonium salts [(Ph_3_P)_2_CPPh_2_]^+^ and [(Ph_3_P)_2_CP­(O)­Ph_2_]^+^, while the relation reverses in case of the dications [(Ph_3_P)_2_CP­(X)­Ph_2_]^2+^.^20d^ With 1.6206(16) Å, the P1–N1 bond is longer than a P=N double bond of a related neutral ylide (1.585(2) Å) but close to its metalated derivative (1.633(2) Å).^20e^ The N1–N2 bond (1.352(2) Å) is shorter than N–N single bonds observed in diaryl-substituted triphenylphosphazines (1.388(5)–1.397(5) Å).^21^ The exocyclic Ga–C1 bond (1.9988(18) Å) resembles values of hydrindacene gallium­(III) compounds reported before.^10e^ Within the cycle, the Ga–C2 bond (1.9278(18) Å) is significantly shorter than the Ga–C3 (1.9911(19) Å) bond, which reflects the different chemical nature of the adjacent carbon atoms. The N2–C3 bond length of 1.313(2) Å agrees will with typical lengths of C=N double bonds for example those in diaryl-substituted triphenylphosphazines (1.309(3)–1.326(7) Å).^21^

Aiming to gain insight into the reaction pathway towards **4**, a conceivable mechanism has been modeled computationally at the ωB97X-D3BJ/def2-TZVP//BP86-D3BJ/def2-TZVP level of theory, Figure 4. Notably, we conducted the computations for two systems, i.e., the Fluind-based scaffold shown in Figure 4 as well as for a simplified phenyl-based framework Figure S29. In both cases, comparable results have been obtained. We considered the formation of a 1:1 adduct as the initial step and **Int1** is formed in an almost thermoneutral process (2.5 kJ mol^–1^). Subsequently, N_2_ loss occurs via **TS1** affording the heterocumulene **Int2**, reflecting the known capability of Ph_3_PCN_2_ to serve as a Ph_3_PC transfer reagent towards ambiphiles.^15^ Notably, the free energy of activation of 112.6 kJ mol^–1^ is much too high to occur under the experimental conditions. Hence, **Int1**, **TS1** as well as **Int2** have also been modeled at the DLPNO-CCSD­(T)/def2-TZVP//BP86-D3BJ/def2-TZVP level of theory and indeed, a significantly lower barrier (59.0 kJ mol^–1^) results, in agreement with the experimental observations. **TS1** is the energetically most demanding step of the overall reaction and hence rate-limiting. Next, **Int2** binds an additional Ph_3_PCN_2_ molecule in an exergonic process (−59.0 kJ mol^–1^) forming **Int3** (see Figure S28), from which C–P bond formation occurs affording the four-membered heterocycle **Int4**. For an animation of the transformation after adduct formation see the Supplementary Data. Cleavage of the adjacent C–P bond gives rise to **Int5** and subsequent migration of a phenyl group of the PPh_3_ unit toward the polarized N_2_CGa carbon takes place via **TS4** providing **Int6** in an irreversible process (−180.0 kJ mol^–1^ relative to **Int5**). Rotation about the Ga–C­(N_2_)­Ph bond (**TS5**) prearranges the N_2_ moiety in a thermoneutral process thus **4** is finally formed traversing through **TS6** in an overall exergonic process (−417.2 kJ mol^–1^). The computational results align well with the experimental observations and highlight the challenges associated with the isolation of a R–Ga=C=PR′_3_ heterocumulene.

**Figure 4:**
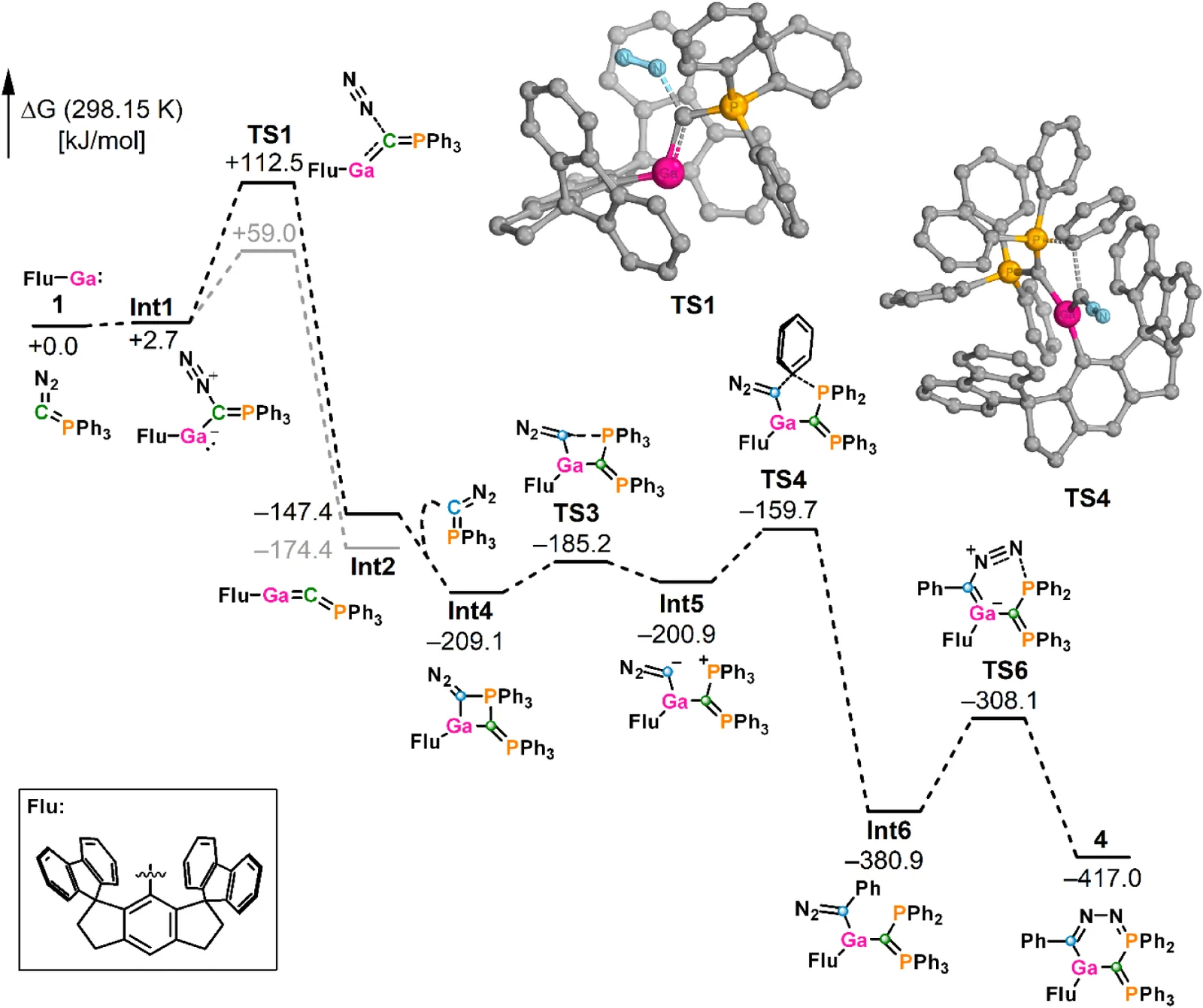
Schematic and simplified potential-energy surface with Gibbs free energies at the ωB97X-D3BJ/def2-TZVP//BP86-D3BJ/def2-TZVP level of theory (black line). For the full energy profile see Figure S28 in the SI. In addition, **Int1**, **TS1** as well as **Int2** have been modeled at the DLPNO-CCSD­(T)/def2-TZVP//BP86-D3BJ/def2-TZVP level of theory (grey line).

## Conclusions

In summary, new reactivity of the gallanediyl **1** with and without the retention of the low oxidation state and singly bonded nature of gallium has been discovered. This highlights the potential of this substance class to undergo diverse reactions, and a fundamental understanding of these processes may help to turn on and off the desired reactivity in future investigations. In here, the carbometalation of cyclohexylallene proceeds with high regioselectivity affording exclusively the 2,3-*syn*-addition product **2**, which contrasts to the reactivity of transition metals that typically give rise to allyl complexes. In the reaction with 1,4-bis­(trimethylsilyl)-1,3-butadiyne, **1** affords selectively the monoinsertion product **3** and hence the second alkyne moiety remains intact for subsequent reactions. Finally, when **1** reacts with the diazophosphorus ylide Ph_3_PCN_2_, an unprecedented C_2_GaN_2_P-heterocycle is formed in a termolecular process. The initial loss of dinitrogen associated with the intermediary formation of a reactive gallium–carbon double is most likely the reason for the divergent reactivity observed in here.

## Experimental Section

### General Information

All preparations were performed under an inert atmosphere of dinitrogen by means of standard Schlenk-line and glovebox (GS Systemtechnik and MBraun) techniques. Prior to usage, all glassware was stored in a 120 °C oven overnight. *n*-Pentane was distilled from LiAlH_4_, benzene was distilled from CaH_2_, toluene was distilled from sodium, and tetrahydrofuran (THF) was distilled from sodium/benzophenone. Benzene-*d*_6_ (99.6%, Sigma-Aldrich) and toluene-*d*_8_ (99.5%, Deutero) were distilled from potassium mirror. All solvents were degassed by three freeze–pump–thaw cycles and stored over activated molecular sieves (4 Å). ^*t*^Bu-M^s^Fluind–Br,^22^ ^*t*^Bu-M^s^Fluind–Li·THF,^10e,22c^ GaCp*,^23^ and Ph_3_PCN_2_ ^15^ were synthesized according to literature procedures. *tert*-Butyllithium (1.7 M in pentane) was purchased from Sigma-Aldrich. 1,4-Bis­(trimethylsilyl)-1,3-butadiyne (98%) was purchased from Sigma-Aldrich and stored at −25 °C. Cyclohexylallene (97%) was purchased from Sigma-Aldrich and stored over activated molecular sieves (4 Å).

**Caution!** The GHS Category 1 corrosive chemical A, carcinogenic chemicals B and C, oxidizing chemical D, and self-reactive chemical E constitute significant safety hazards and must be handled with extreme care.

**Caution!** Extreme care should be taken both in the handling of cryogen liquid nitrogen and its use in the Schlenk line trap to avoid the condensation of oxygen from air.

**Caution!** *tert*-Butyllithium is extremely pyrophoric. It must be handled using proper needle and syringe techniques.

### Characterization

^1^H and ^13^C chemical shifts are referenced to the residual solvent signals of benzene-*d*_6_ (^1^H, 7.16 ppm; ^13^C, 128.06 ppm). The chemical shifts δ are reported in [ppm] and the coupling constants *J* are given in Hertz [Hz]. NMR experiments were performed at room temperature (298 K) using a Bruker Avance III-500 MHz spectrometer. Assignment of the signals: s = singlet, d = doublet, t = triplet, q = quartet, m = multiplet, br = broad signal, dd = doublet of doublets, dt = doublet of triplets. Diffusion-ordered spectroscopy was conducted by Dr. Janine Kowalke.

Elemental analyses were performed on site by Khrystyna Gerlach and Jana Buschmann using a Vario MICRO cube from Elementar Analysensysteme GmbH. The samples were prepared under an inert nitrogen glovebox atmosphere and loaded in a tin boat. The combustion of the substance takes place in a combustion tube at 1150 °C in a helium atmosphere enriched with oxygen. During combustion, the gaseous products CO_2_, H_2_O, N_2_, NO, NO_2_, SO_2_, and SO_3_ are formed from C, H, N, and S, respectively. In a downstream reduction tube (filled with wire-shaped copper), the nitrogen oxides and sulfur oxides are reduced to N_2_ and SO_2_. The gas mixture is separated in U-shaped separation columns on special adsorbents. After separation, the gases are quantitatively detected with a thermal conductivity detector (TCD). In cases of **2** and **3**, the experimental values are low on carbon, which points to incomplete combustion, trapped nonvolatile carbon, or the presence of moisture and other volatile impurities. IR spectra (given in cm^–1^) were recorded with an Agilent Cary 630 FT-IR spectrometer using a diamond ATR unit inside a nitrogen-filled glovebox.

Spectroscopic Raw Data Are Available via the Repository Zenodo.^24^ Synthesis of **2**: Inside a glovebox, a J. Young NMR tube was charged with a solution of ^*t*^Bu-M^s^Fluind–Li·THF (30 mg, 0.0367 mmol, 1.0 equiv) in benzene-*d*_6_ (0.45 mL). A solution of GaCp* (7.5 mg, 0.0367 mmol, 1.0 equiv) in benzene-*d*_6_ (0.15 mL) was added to the NMR tube and heated at 70 °C for 16 h to give a sticky yellow mixture. Cyclohexylallene (4.5 mg, 0.0367 mmol, 1.0 equiv) was added and the NMR tube was heated to 50 °C for 16 h. Subsequently, filtration gave a yellow solution and slow evaporation of the solvent afforded yellow crystals, which were isolated, washed with *n*-pentane (2 × 1 mL) and dried *in vacuo* to give **2** (18.4 mg, 0.0198 mmol, 54%) as a yellow solid. Despite multiple attempts **2** remained contaminated with the proteo-ligand ^*t*^Bu-M^s^Fluind–H (ca. 25%, see Figures S4 and S5). The formation of M^s^Fluind–H as an unavoidable side product has been described recently by Beckmann and coworkers.^16^

^**1**^ **H NMR** (500 MHz, C_6_D_6_, ppm): δ = 7.62 (br, 2H, H^Ar^), 7.51 (d, *J* = 8.0 Hz, 1H, H^Ar^), 7.47 (dd, *J* = 8.0, 2.6 Hz, 4H, H^Ar^), 7.37–7.33 (m, 2H, H^Ar^), 7.28–7.23 (m, 3H, H^Ar^), 7.18 (br, 1H, H^Ar^), 3.82 (dt, *J* = 7.2, 2.7 Hz, 1H, H^allyl^), 2.66 (d, *J* = 14.1 Hz, 2H, CH_2_), 2.43 (d, *J* = 14.1 Hz, 2H, CH_2_), 1.99 (d, *J* = 2.8 Hz, 2H, CH_2_^allyl^), 1.70 (s, 6H, CH_3_), 1.55 (s, 6H, CH_3_), 1.40 (s, 18H, ^*t*^Bu), 1.25 (s, 18H, ^*t*^Bu), 1.13 (m, 4H, H^Cy^), 1.03 (m, 4H, H^Cy^), 0.69 (m, 3H, H^Cy^).

^**13**^ **C­{** ^**1**^ **H} NMR** (126 MHz, C_6_D_6_, ppm): δ = 185.8 (C_3_–Ga), 156.0 (C^Ar^), 155.2 (C^Ar^), 154.5 (C^Ar^), 151.0 (C^Ar^), 150.8 (C^Ar^), 142.8 (C^Ar^), 138.6 (C^Ar^), 138.1 (C^Ar^), 137.8 (C^Ar^), 135.5 (C^vinyl^), 125.9 $\left(C_{H}^{\mathrm{Ar}}\right)$ , 124.2 $\left(C_{H}^{\mathrm{Ar}}\right)$ , 121.3 $\left(C_{H}^{\mathrm{Ar}}\right)$ , 120.4 $\left(C_{H}^{\mathrm{Ar}}\right)$ , 120.3 $\left(C_{H}^{\mathrm{Ar}}\right)$ , 119.4 $\left(C_{H}^{\mathrm{Ar}}\right)$ , 115.1 $\left(C_{H}^{\mathrm{Ar}}\right)$ , 63.6 (C_16,21_), 57.9 $\left(C_{17,22}^{{\mathrm{CH}}_{2}}\right)$ , 43.0 $\left(C_{18,23}^{\mathrm{Me}\left(q\right)}\right)$ , 36.1 $\left(C_{{\mathrm{CH}}_{2}}^{\mathrm{Cy}}\right)$ , 35.5 (C50,54,58,62But(q)) , 35.0 (C50,54,58,62But(q)) , 33.9 $\left(C_{19,20-24,25}^{\mathrm{Me}}\right)$ , 33.1 $\left(C_{\mathrm{CH}}^{\mathrm{Cy}}\right)$ , 32.9 $\left(C_{{\mathrm{CH}}_{2}}^{\mathrm{Cy}}\right)$ , 32.7 $\left(C_{19,20-24,25}^{\mathrm{Me}}\right)$ , 31.8 (CMeBut) , 29.1 $\left(C_{H_{2}A,H_{2}B}^{\mathrm{Fluind}-{\mathrm{CH}}_{2}-\mathrm{CGa}}\right)$ , 26.9 $\left(C_{{\mathrm{CH}}_{2}}^{\mathrm{Cy}}\right)$ , 26.4 $\left(C_{{\mathrm{CH}}_{2}}^{\mathrm{Cy}}\right)$ .

**Elemental Analysis** calcd (%) for C_65_H_79_Ga: C 83.94, H 8.56; found: C 83.16, H 8.61.

Synthesis of **3**: Inside a glovebox, a J. Young NMR tube was charged with a solution of ^*t*^Bu-M^s^Fluind–Li·THF (30 mg, 0.0367 mmol, 1.0 equiv) in benzene-*d*_6_ (0.45 mL). A solution of GaCp* (7.5 mg, 0.0367 mmol, 1.0 equiv) in benzene-*d*_6_ (0.15 mL) was added to the NMR tube and heated at 70 °C for 16 h to give a sticky yellow mixture. 1,4-Bis­(trimethylsilyl)-1,3-butadiyne (7.14 mg, 0.0367 mmol, 1.0 equiv) was added and the NMR tube was rotated for 16 h at room temperature. Subsequent filtration afforded an orange solution and slow evaporation of the solvent gave orange crystals, which were isolated, washed with *n*-pentane (2 × 1 mL) and dried *in vacuo* to give **3** (23 mg, 0.0229 mmol, 62%) as an orange solid.

^**1**^ **H NMR** (500 MHz, C_6_D_6_, ppm): δ = 7.96 (br, 2H, H^Ar^), 7.55 (d, *J* = 8.0 Hz, 2H, H^Ar^), 7.45 (d, *J* = 7.9 Hz, 2H, H^Ar^), 7.31–7.28 (m, 5H, H^Ar^), 7.25 (d, *J* = 8.0 Hz, 2H, H^Ar^), 2.46 (d, *J* = 14.2 Hz, 2H, CH_2_), 2.42 (d, *J* = 14.4 Hz, 2H, CH_2_), 1.55 (s, 12H, 4xCH_3_), 1.40 (s, 18H, ^*t*^Bu), 1.33 (s, 18H, ^*t*^Bu), 0.05 (s, 9H, TMS), −0.25 (s, 9H, TMS).

^**13**^ **C­{** ^**1**^ **H} NMR** (126 MHz, C_6_D_6_, ppm): δ = 199.1 (C_1_–Ga), 157.3 (C^Ar^), 155.4 (C^Ar^), 155.3 (C^Ar^), 150.3 (C^Ar^), 149.7 (C^Ar^), 140.6 (C^Ar^), 138.5 (C^Ar^), 137.7 (C^Ar^), 135.1 (C^Ar^), 134.6 (C^olefin^), 125.9 $\left(C_{H}^{\mathrm{Ar}}\right)$ , 124.0 $\left(C_{H}^{\mathrm{Ar}}\right)$ , 121.8 $\left(C_{H}^{\mathrm{Ar}}\right)$ , 121.3 $\left(C_{H}^{\mathrm{Ar}}\right)$ , 119.6 $\left(C_{H}^{\mathrm{Ar}}\right)$ , 119.4 $\left(C_{H}^{\mathrm{Ar}}\right)$ , 117.3 $\left(C_{H}^{\mathrm{Ar}}\right)$ , 105.2 $\left(C_{3-4}^{\mathrm{Alkyne}}\right)$ , 94.9 $\left(C_{3-4}^{\mathrm{Alkyne}}\right)$ , 63.5 (C_13,25_), 60.3 $\left(C_{14,24}^{{\mathrm{CH}}_{2}}\right)$ , 42.1 $\left(C_{15,21}^{\mathrm{Me}\left(q\right)}\right)$ , 35.4 (C30,41,50,61But(q)) , 35.0 (C30,41,50,61But(q)) , 33.4 $\left(C_{16,17-22,23}^{\mathrm{Me}}\right)$ , 32.7 $\left(C_{16,17-22,23}^{\mathrm{Me}}\right)$ , 32.1 (CMeBut) , 31.7 (CMeBut) , 1.0 (C^TMS^), 0.3 (C^TMS^).

^**29**^ **Si DEPT135 NMR** (99 MHz, C_6_D_6_, ppm): δ = −13.6 (TMS), −21.1 (TMS).

**Elemental Analysis** calcd (%) for C_66_H_83_GaSi_2_: C 79.09, H 8.35; found: C 77.60, H 8.11.

Synthesis of **4**: Inside a glovebox, a J. Young NMR tube was charged with a solution of ^*t*^Bu-M^s^Fluind–Li·THF (30 mg, 0.0367 mmol, 1.0 equiv) in benzene-*d*_6_ (0.45 mL). A solution of GaCp* (7.5 mg, 0.0367 mmol, 1.0 equiv) in benzene-*d*_6_ (0.15 mL) was added to the NMR tube and heated at 70 °C for 16 h to give a sticky yellow mixture. Ph_3_PCN_2_ (22.2 mg, 0.0734 mmol, 2.0 equiv) was suspended in benzene-*d*_6_ (0.15 mL), added to the NMR tube causing an immediate color change to orange and the formation of gas bubbles (most likely N_2_) was observed. The NMR tube was rotated for 16 h at room temperature and then the solids were filtered off, which gave an orange solution. Slow evaporation of the solvent afforded orange crystals, which were isolated, washed with *n*-pentane (2 × 1 mL) and dried *in vacuo* to give **4** (33.3 mg, 0.0235 mmol, 64%) as an orange solid.

^**1**^ **H NMR** (500 MHz, C_6_D_6_, ppm): δ = 7.73 (d, *J* = 8.0 Hz, 2H, H^Ar^), 7.69 (d, *J* = 1.6 Hz, 2H, H^Ar^), 7.67 (s, 1H, H^Ar^), 7.62 (dd, *J* = 8.0, 1.7 Hz, 2H, H^Ar^), 7.34–7.21 (m, 12H, H^Ar^), 7.13–7.09 (m, 2H, H^Ar^), 7.01–6.96 (m, 4H, H^Ar^), 6.96–6.90 (m, 6H, H^Ar^), 6.90–6.81 (m, 5H, H^Ar^), 6.79–6.71 (m, 5H, H^Ar^), 6.31–6.26 (m, 2H, H^Ar^), 2.56 (d, *J* = 13.6 Hz, 2H, CH_2_), 1.86 (d, *J* = 13.7 Hz, 2H, CH_2_), 1.76 (s, 6H, CH_3_), 1.58 (s, 6H, CH_3_), 1.28 (s, 18H, ^*t*^Bu), 1.20 (s, 18H, ^*t*^Bu).

^**13**^ **C­{** ^**1**^ **H} NMR** (126 MHz, C_6_D_6_, ppm): δ = 155.8 (C^Ar^), 155.6 (C^Ar^), 152.4 (C^Ar^), 149.6 (C^Ar^), 149.5 (C^Ar^), 148.6 (C^Ar^), 139.9 (C^Ar^), 138.0 (C^Ar^), 136.4 (C^Ar^), 134.5 (dd, *J*_*C,P*_ = 60.8, 9.2 Hz, P–C^57^–P), 133.7 (d, *J*_*C,P*_ = 10.5 Hz, P­(C_6_H_5_)), 133.5 (d, *J*_*C,P*_ = 7.2 Hz, P­(C_6_H_5_)), 131.6 (d, *J*_*C,P*_ = 9.7 Hz, P­(C_6_H_5_)), 130.4 $\left(C_{H}^{\mathrm{Ar}}\right)$ , 129.7 $\left(C_{H}^{\mathrm{Ar}}\right)$ , 129.0 (d, *J*_*C,P*_ = 2.9 Hz, P­(C_6_H_5_)), 126.6 $\left(C_{H}^{\mathrm{Ar}}\right)$ , 126.5 $\left(C_{H}^{\mathrm{Ar}}\right)$ , 126.4 $\left(C_{H}^{\mathrm{Ar}}\right)$ , 124.3 $\left(C_{H}^{\mathrm{Ar}}\right)$ , 124.2 $\left(C_{H}^{\mathrm{Ar}}\right)$ , 121.9 $\left(C_{H}^{\mathrm{Ar}}\right)$ , 119.7 $\left(C_{H_{4}}^{\mathrm{Ar}}\right)$ , 119.4 (d, *J*_*C,P*_ = 8.2 Hz, P­(C_6_H_5_)), 68.1 (C_7,12_), 62.9 $\left(C_{8,13}^{{\mathrm{CH}}_{2}}\right)$ , 42.1 $\left(C_{9,14}^{\mathrm{Me}\left(q\right)}\right)$ , 36.3 $\left(C_{10,11-15,16}^{\mathrm{Me}}\right)$ , 35.4 (C29,33,49,53But(q)) , 35.1 (C29,33,49,53But(q)) , 31.7 (CMeBut) , 31.1 (CMeBut) , 31.0 $\left(C_{10,11-15,16}^{\mathrm{Me}}\right)$ .

^**31**^ **P­{** ^**1**^ **H} NMR** (203 MHz, C_6_D_6_, ppm): δ = 6.1 (d, ^2^ *J*_*P,P*_ = 53.7 Hz), −6.0 (d, ^2^ *J*_*P,P*_ = 53.7 Hz).

**Elemental Analysis** calcd (%) for C_96_H_103_GaN_2_P_2_: C 81.40, N 1.98, H 7.33; found: C 81.35, N 2.24, H 6.92.

**IR** (ATR diamond, cm^–1^): 3050, 2955, 2903, 2863, 1588, 1480, 1438, 1384, 1359, 1253, 1229, 1180, 1129, 1095, 1068, 1028, 1002, 975, 926, 898, 884, 829, 818, 786, 742, 710, 691, 622, 594, 557, 539, 526.

### Computational Details

All geometry optimizations and frequency calculations were performed using ORCA 6.1.1 ^25^ employing the BP86 ^26^ functional with def2-TZVP basis sets.^27^ To account for dispersion effects, Grimme’s D3 dispersion correction along with Becke-Johnson damping was used.^28^ To confirm the identity of stationary states, vibrational frequency calculations were carried out. All minima were absent of negative frequencies and transition states had one imaginary frequency corresponding to the reaction coordinate. To check if the transition states connect the related intermediates, the intrinsic reaction coordinate (IRC) was calculated. Thermochemical calculations to obtain Gibbs free energies and enthalpies were carried out at standard conditions (298.15 K, 1 atm).

Single-point calculations were carried out using the ωB97X-D3BJ^29^ range-separated hybrid functional with the def2-TZVP basis sets.^27^ Herein, solvent effects were included implicitly with the SMD solvation model for benzene.^30^

To accurately describe the N_2_ dissociation step, a single-point DLPNO-CCSD­(T)^31^ calculation with def2-TZVP basis sets^27^ of the transition state and the connected intermediates was carried out. For the visualization of geometries, IboView was used.^32^

XYZ-coordinates of the geometry optimized structures are available via the repository Zenodo.^24^

## Supplementary Material







## Notes

Mechanism for the formation of **4** (GIF)
Video presentation of Ga Mechanism (MP4)
Additional experimental, crystallographic, and computational details as well as NMR and IR spectra (PDF)

## Abbreviations

Cy: cyclohexyl
DOSY: diffusion ordered NMR spectroscopy
IR: infrared
NMR: nuclear magnetic resonance
sc-XRD: single-crystal X-ray diffraction
TMS: trimethylsilyl
