# Supplementary material for: Redox-Divergent Reactivity of a Gallium(I) Ambiphile toward an Allene, a Diyne, and Ph3PCN2
Source: Inorg Chem. 2026 Aug 10;65(33):19548–56. doi: 10.1021/acs.inorgchem.6c03200 (PMC13509014; doi:10.1021/acs.inorgchem.6c03200)
Supplement: Supplementary file 1 [file ic6c03200_si_003.pdf]

Supporting Information

Redox-Divergent Reactivity of a Gallium(I) Ambiphile Towards an Allene, a Diyne, and Ph_3PCN_2

Simon H. F. Schreiner,[a] Jan N. Ludwig,[b] Tobias Rüffer,[a] Max M. Hansmann,[b] and Robert Kretschmer*[acd]*

a) Dr. S. H. F. Schreiner, Dr. T. Rüffer, Prof. Dr. R. Kretschmer, Institut für Chemie, Technische Universität Chemnitz, Strasse der Nationen 62, 09111 Chemnitz, Germany

b) J. N. Ludwig, Prof. Dr. M. M. Hansmann, Technische Universität Dortmund, Otto-Hahn-Straße 6, 44227 Dortmund, E-mail: max.hansmann@tu-dortmund.de

c) Prof. Dr. R. Kretschmer, Jena Center of Soft Matter, Friedrich-Schiller-Universität Jena, Philosophenweg 7, 07443 Jena, Germany, E-mail: robert.kretschmer@chemie.tu-chemnitz.de

Table of contents

1. Additional Experimental Details....3
2. Crystallographic Details....13
3. Spectra....20
4. Additional computational data....32
5. References....34

1. Additional Experimental Details

Reaction of ^{t}Bu-M^{s}Fluind–Ga **1** with Ph_3PCN_2 in 1:1 stoichiometry

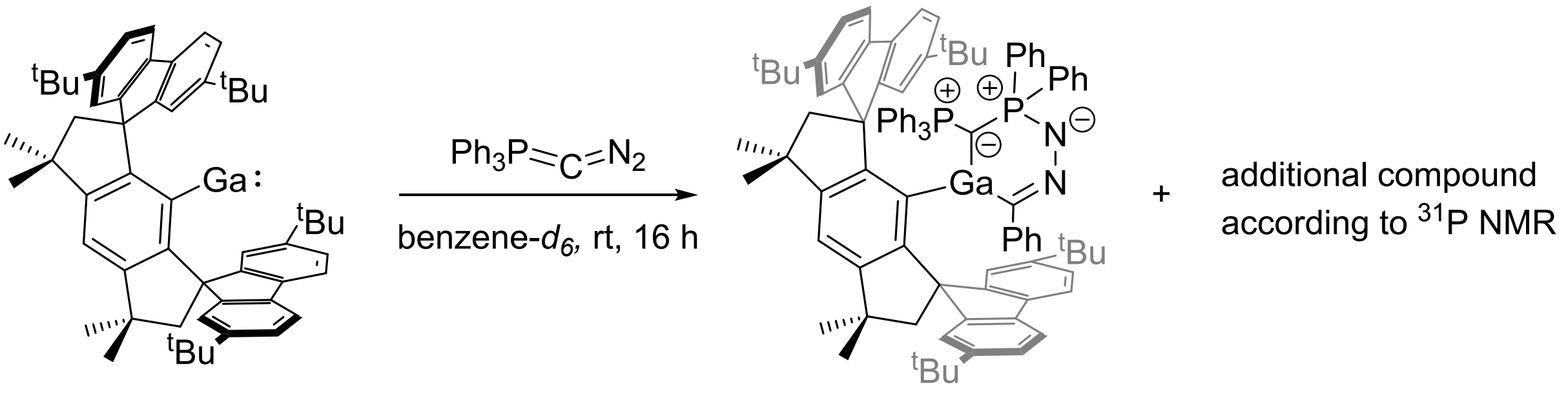

1 **4**

Inside a glovebox, a J. Young NMR tube was charged with a solution of ^{t}Bu-M^{s}Fluind–Li·THF (30 mg, 0.0367 mmol, 1.0 equiv.) in benzene-d_6 (0.45 mL). A solution of GaCp* (7.5 mg, 0.0367 mmol, 1.0 equiv.) in benzene-d_6 (0.15 mL) was added to the NMR tube and heated at 70 ˚C for 16 hours to give a sticky yellow mixture. Ph_3PCN_2 (11.1 mg, 0.0367 mmol, 1.0 equiv.) was suspended in benzene-d_6 (0.15 mL) and added to the NMR tube causing an immediate color change to orange.

The $^{31}P\{^1H\}$ NMR spectrum showed signals of the product compound **4** at 6.1 ppm (d, $^2J_{P,P}$ = 53.7 Hz) and −6.0 ppm (d, $^2J_{P,P}$ = 53.7 Hz), and an additional signal at −10.3 (s) ppm (see Figures S1 and S2).

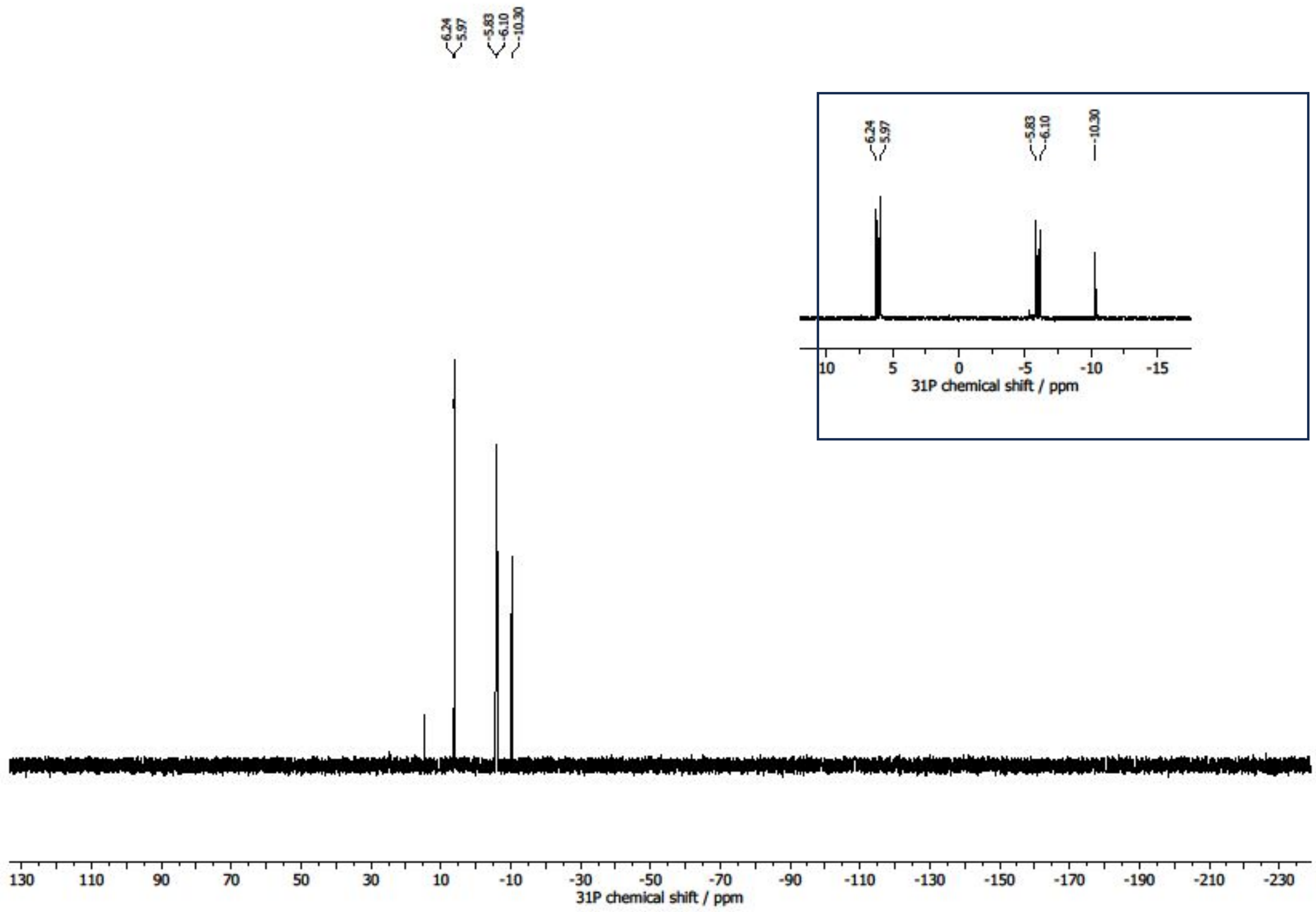

Figure S1. $^{31}P\{^{1}H\}$ NMR spectrum (203 MHz, C_6D_6, 298 K) of the reaction of ^{t}Bu-M^{s}Fluind–Ga **1** with Ph_3PCN_2 in a 1:1 stoichiometry.

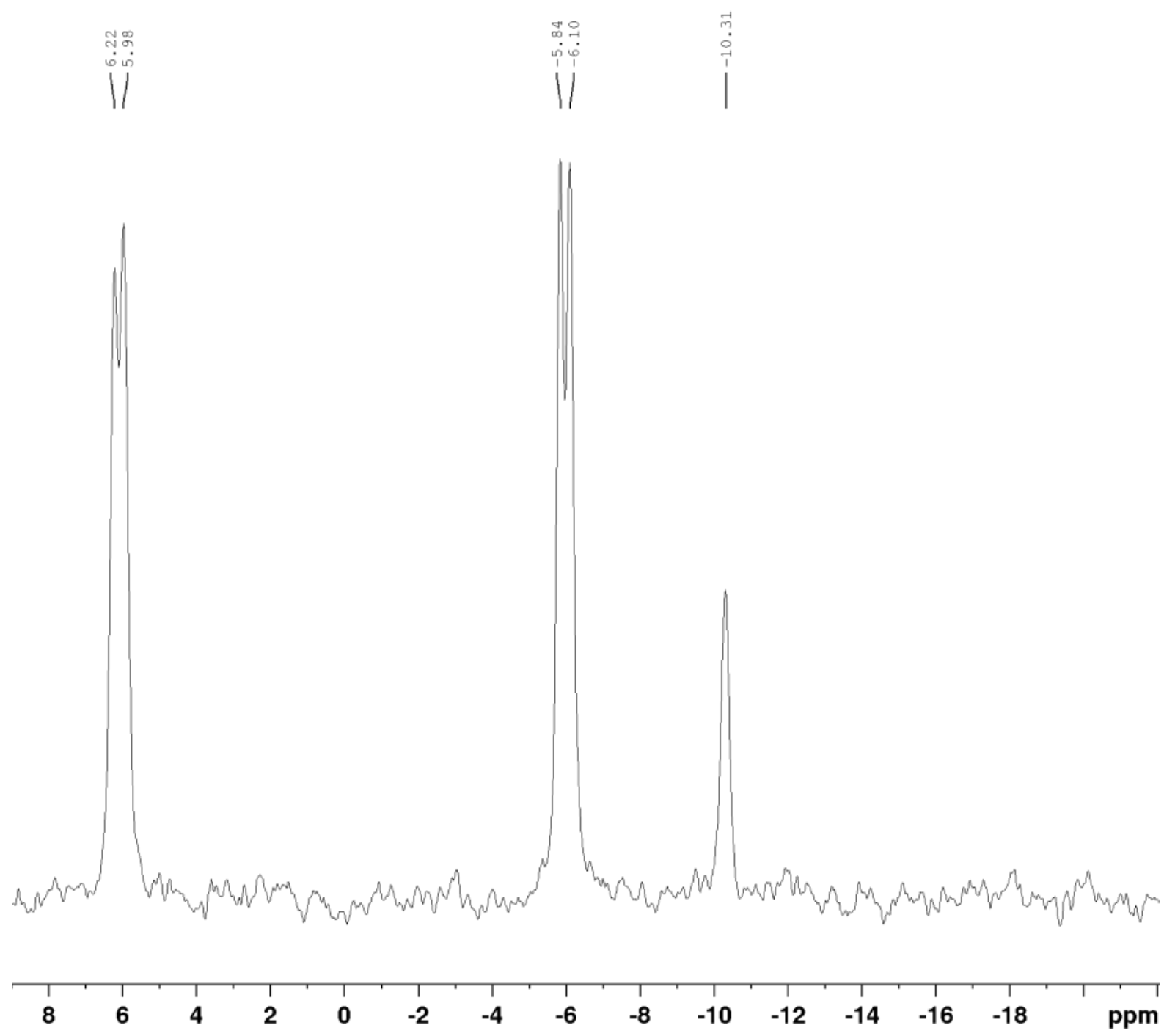

Figure S2. $^{31}P\{^{1}H\}$ NMR spectrum of the reaction mixture of gallanediyl **1** with Ph_3PCN_2 in a 1:1 stoichiometry in C_6D_6 with a relaxation delay (d1) of 5 s. Owing to a relatively low signal-to-noise ratio, the dataset was processed using an exponential apodization corresponding to a line broadening of 20 Hz.

The nature of the additional compound was tried to evaluate by crystallization, however the only compounds observed as single-crystals were ^{t}Bu-M^{s}Fluind–Ga **1** (yellow crystals) and product **4** (orange crystals). No other sort of crystals could be visually identified under the microscope and the evaluation of several crystals by cell measurements revealed either **1** or **4**. Moreover, recording a $^{31}P\{^{1}H\}$ NMR spectrum of the supernatant surprisingly showed no signal at −10.3 (s) ppm anymore.

To evaluate this further, a ^{31}P DOSY NMR spectrum of a fresh reaction mixture with an optimized d_1 time of five seconds was recorded (see Figure S3). The DOSY NMR experiment was performed with the standard Bruker ledbpgp2s DOSY pulse program and parameters were optimized for ^{31}P DOSY. The data were processed and analyzed using the Dynamics Center software package. Details are given in the following.

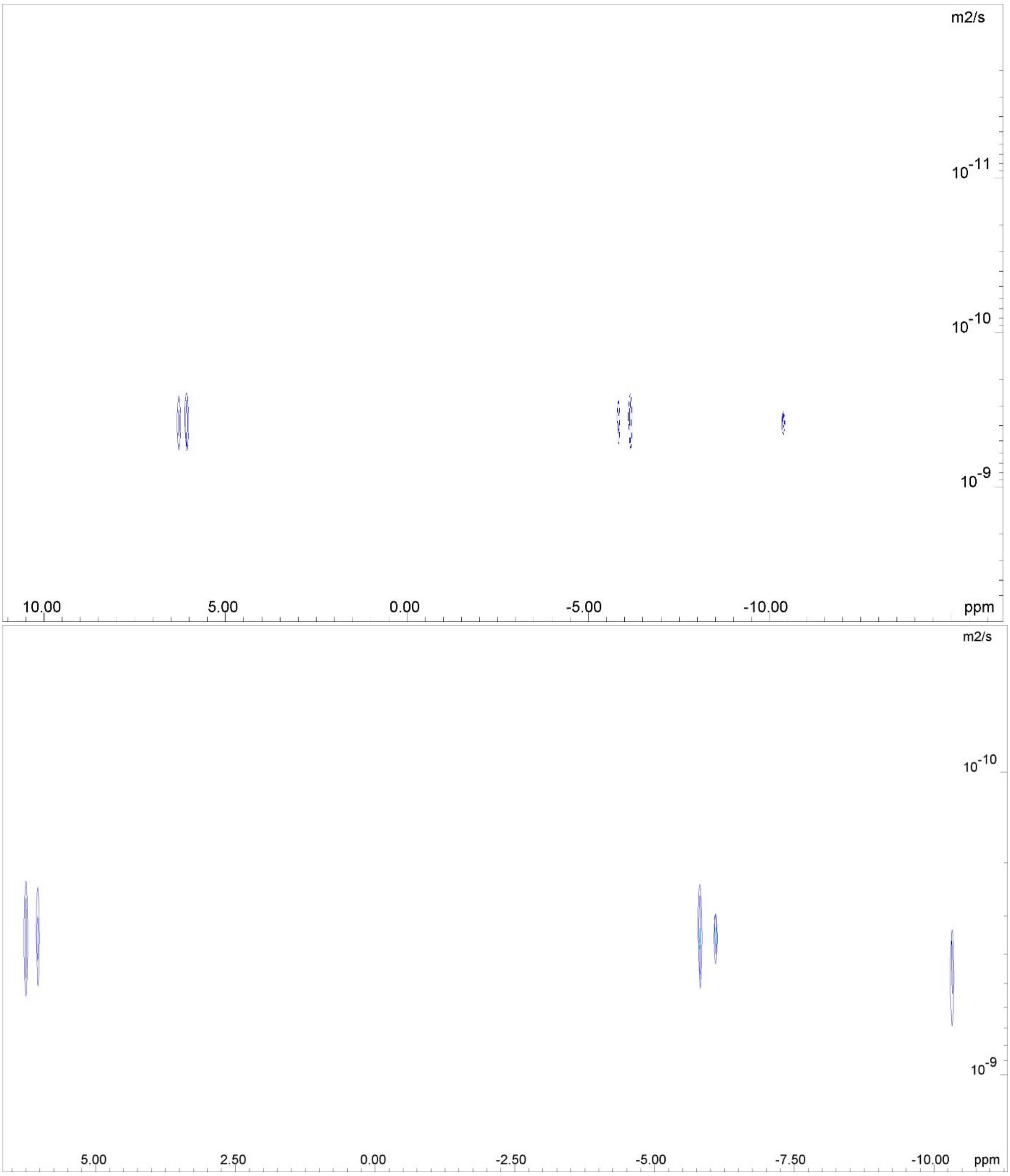

Figure S3. ^{31}P DOSY NMR spectrum in two different ranges. DOSY experiment acquired in C_6D_6 at 298 K. Gradient pulses (p30) were set to 2 ms with a diffusion delay (d20) $\Delta = 90$ ms. Gradient strengths were incremented linearly from 2 to 95 % of the G(P)U-6 scale in 16 steps.

The diffusion behavior of molecular species in solution is commonly interpreted using the Stokes–Einstein (SE) equation (1)[1], which relates the diffusion coefficient D to temperature T, solvent viscosity η, and the hydrodynamic radius r_H of the solute. However, the classical SE relation performs poorly for small organic molecules below approximately 0.5–1 kDa, since its underlying assumptions - treating solutes as hard spherical particles diffusing in a continuum solvent - no longer hold at molecular dimensions. Under such conditions, SE systematically underestimates diffusion coefficients, as the discrete and anisotropic nature of molecular solutes invalidate continuum hydrodynamics. The SE equation is therefore only reliable for large solutes such as peptides, polymers, micelles, or nanoparticles (typically above 1–2 kDa), where an approximately spherical hydrodynamic radius (2) and continuum behavior become valid assumptions.[2]

$$D = \frac{k_B T}{6\pi f \eta r_H} \tag{1}$$

$$r_H = \left(\frac{3M_W}{4\pi N_A \rho_{eff}}\right)^{\frac{1}{3}} \tag{2}$$

$$M_W = \frac{4\pi}{3} N_A \rho_{eff} \left(\frac{k_B T}{6\pi\eta f D}\right)^3 \tag{3}$$

Table S1. Theoretical molar mass calculated from diffusion coefficient using the SE method (equation 1-3).

Signal	δ [ppm]	10^{-10} D [m^2 s^{-1}]	M_{cal} [g mol^{-1}]	M_{theo} [g mol^{-1}]
1	6.22	3.60	1322	1384
2	5.97	3.95	1001	

3	−5.86	3.80	1124	
4	−6.11	3.61	1311	
5	−10.33	4.14	869	1110

$k_B = 1.380649\ 10^{-23}$ J K^{-1} $\rho_{eff} = 619$ kg m^{-3}[2] $\eta = 6.4085\ 10^{-4}$ kg m^{-1} s^{-1}[2b]
$N_A = 6.02214076\ 10^{23}$ mol^{-1} f = 1 (for sphere)

The experimentally measured diffusion coefficients were used to calculate the molar masses (3) of the studied molecules. Using the Stokes–Einstein (SE) equation, the calculated molar masses show good agreement with the theoretical values, indicating that SE provides a reliable approximation in this case. This agreement relies on several key assumptions: the molecules are treated as rigid, spherical particles (f = 1), the solvent is considered a continuous homogeneous medium, and effects of molecular flexibility, anisotropic shape, or specific solute–solvent interactions are neglected. Moreover, the effective density (ρ_{eff}) of the solute and the solvent viscosity (η) were taken from the literature; these values were determined specifically for small organic molecules.

As a second approach for molar mass determination, DOSY NMR diffusion data were evaluated using the universal calibration model developed by Hiller *et al.*[3] While hydrodynamic radii and molar masses can in principle be estimated from diffusion coefficients via the Stokes–Einstein equation, this approach often suffers from significant inaccuracies for non-spherical molecules, flexible structures, aggregates, and systems with poorly defined solvation shells. Consequently, the Stokes–Einstein-based molar mass estimates obtained in this work should be regarded only as rough approximations. To achieve a more reliable determination of molar masses, the universal DOSY calibration proposed by Hiller *et al.* was employed.[3] This model establishes a direct relationship between diffusion coefficients and molar masses that is

independent of molecular structure, solvent, and temperature and has been validated over a molecular-weight range spanning several orders of magnitude. Furthermore, the concept of external calibration curves (ECCs), as introduced by Stalke and co-workers,[4] demonstrates the applicability of DOSY-derived diffusion coefficients for accurate molecular-weight estimation in common NMR solvents.

All molar mass calculations were performed using the equations, calibration parameters, and empirical constants reported by Hiller *et al.*[3] and Stalke and co-workers.[5,4] The experimentally obtained diffusion coefficients were evaluated according to these published methodologies to derive the corresponding molar masses. In addition, the calculations were carried out using the different molecular shape models proposed in the universal calibration approach, namely compact spheres (CS), dissipated spheres and ellipsoids (DSE), and expanded discs (ED), allowing the influence of molecular geometry on the calculated molar masses to be assessed.

Table S2. Theoretical molar mass calculated from diffusion coefficient using equations, parameters for C_6D_6 reported from Hiller.[3]

Signal	δ [ppm]	10^{-10} D [$m^2\ s^{-1}$]	M_{cal} [g mol^{-1}]			M_{theo} [g mol^{-1}]
			CS	DSE	ED	
1	6.22	3.60	3196	1475	1097	1384
2	5.97	3.95	2621	1273	971	
3	−5.86	3.80	2848	1353	1022	
4	−6.11	3.61	3178	1468	1093	
5	−10.33	4.14	2371	1181	913	1110

$$\log(MW) = -\frac{1}{\propto +1}\left(3.0333 \log\left(\frac{\eta D}{T}\right) + \log(K) + 42.1101\right)$$

η = 0.00063814 Pa s, CS: α = 0.41888, K = 0.057471; DSE: α = 0.90806, K = 0.0048527; ED: α = 1.3137, K = 0.00049889; T = 298 K

Table S3. Theoretical molar mass calculated from diffusion coefficient using equations, parameters for C_6D_6 reported from Stalke.[5]

Signal	δ [ppm]	10^{-10} D [m^2 s^{-1}]	M_{cal} [g mol^{-1}]			M_{theo} [g mol^{-1}]
			CS	DSE	ED	
1	6.22	3.60	2820	1490	1069	1384
2	5.97	3.95	2331	1283	950	
3	−5.86	3.80	2524	1366	998	
4	−6.11	3.61	2804	1483	1065	
5	−10.33	4.14	2117	1190	895	1110

$$MW = 10^{\left(\frac{\log D - \log K}{\alpha}\right)}$$

CS: α = -0.488, log K = -7.76; DSE: α = -0.622, log K = -7.47; ED: α = -0.787, log K = -7.06

Among the applied shape models, the best agreement was obtained with the DSE (dissipated spheres and ellipsoids) parameter for which the corresponding molar masses showed a deviation of only max. 3% from the expected values.

Ph_3PCN_2 features high electron density at carbon and nitrogen and hence nucleophilic behavior is expected at both atoms.[6] In fact, related stable diazoalkenes (R_2C=C=N_2) have been shown to react with the boron Lewis acid $B(C_6F_5)_3$ to form adducts via carbon and nitrogen depending on the reaction conditions. Thus, in agreement with the experimentally obtained diffusion coefficient from the ^{31}P DOSY measurement and the calculated molar mass, two (**5** and **5'**) initial Lewis-acid (empty p orbital of ^{t}Bu-M^{s}Fluind–Ga) Lewis-base (lone pair Ph_3PCN_2) adducts are postulated. While computations (see the main article) indicate that **5** should undergo rather fast N_2 loss, **5'** might represent a kinetically trapped state and could be assigned to the compound detected at −10.3 (s) ppm in the ^{31}P NMR spectrum.

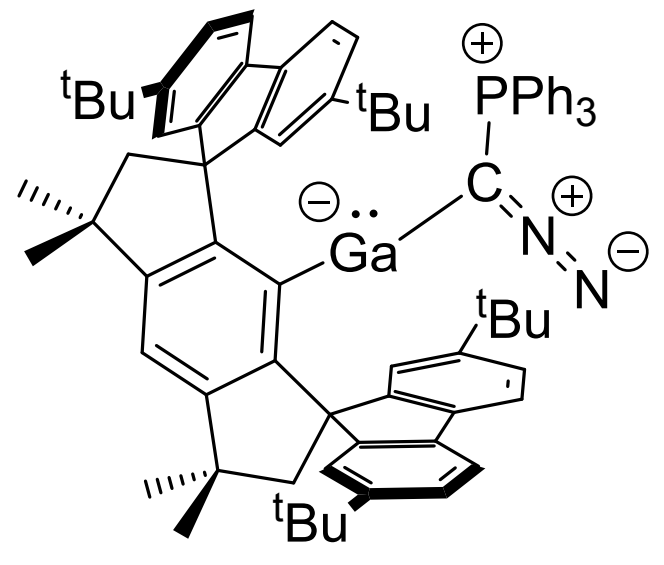

5
1110.18 g mol^{-1}

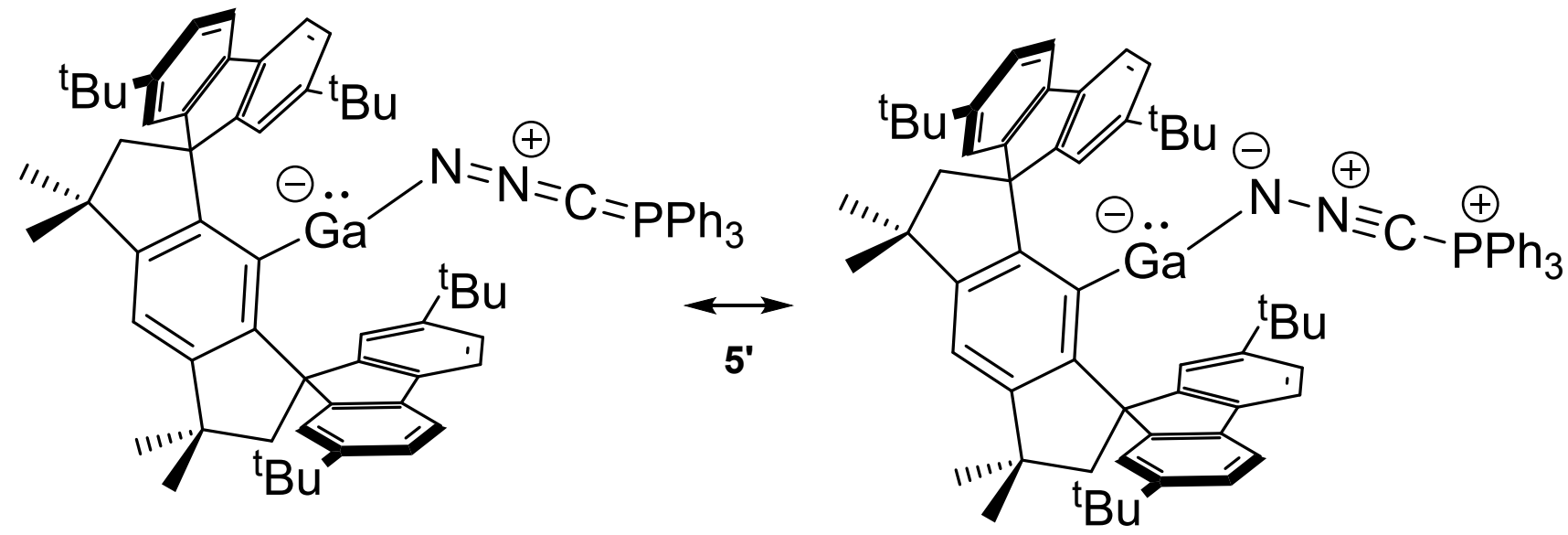

5'

To gain more insight into the formation of both compounds, low-temperature NMR measurements were conducted: Inside a glovebox, a J. Young NMR tube was charged with a solution of ^{t}Bu-M^{s}Fluind–Li·THF (30 mg, 0.0367 mmol, 1.0 equiv.) in toluene-d_8 (0.45 mL). A solution of GaCp* (7.5 mg, 0.0367 mmol, 1.0 equiv.) in toluene-d_8 (0.15 mL) was added to the NMR tube and heated at 70 °C for 16 hours to give a sticky yellow mixture. On the Schlenk line, a solution of Ph_3PCN_2 (11.1 mg, 0.0367 mmol, 1.0 equiv.) in toluene-d_8 (0.15 mL) was cooled down to −75 °C added to the cooled (−75 °C) NMR tube. The NMR tube was immediately transferred to the NMR spectrometer and $^{31}P\{^1H\}$ NMR spectra were recorded at −75 °C, and the tube was warmed up in 10 °C increments up to 25 °C (see Figure S4).

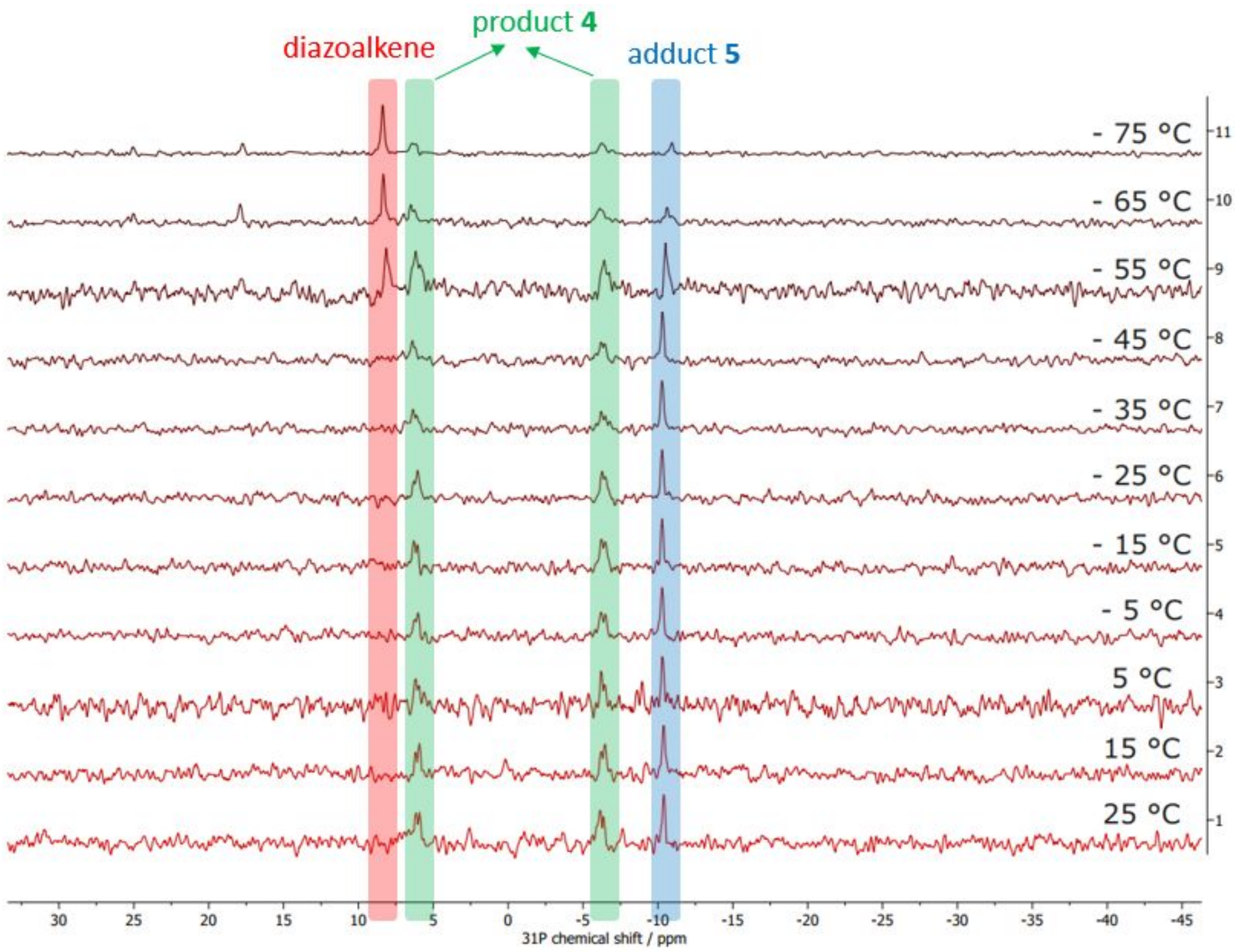

Figure S4. Low-temperature $^{31}P\{^1H\}$ NMR (203 MHz, toluene-d_8) measurements of the reaction of ^{t}Bu-M^{s}Fluind–Ga **1** with Ph_3PCN_2 in a 1:1 stoichiometry.

It was observed that the formation of product **4** and the intermediate occured even at −75 ˚C. By reaching the temperature of −45 ˚C, the starting material had been fully consumed. Therefore, it was not possible to form one compound exclusively by temperature restrictions.

To gain additional insight, we conducted a low-temperature (-60 ˚C) *in situ* IR experiment, Figure S5. At the beginning of the experiment, the distinct vibration of Ph_3PCN_2 can be recognized at 1995 cm^{-1}. The signal decays over time but no additional band in account of a cumulene can be recognized. This argues against the formation of **5** or **5’** mentioned above and hints towards either **6** or another yet unknown side-product.

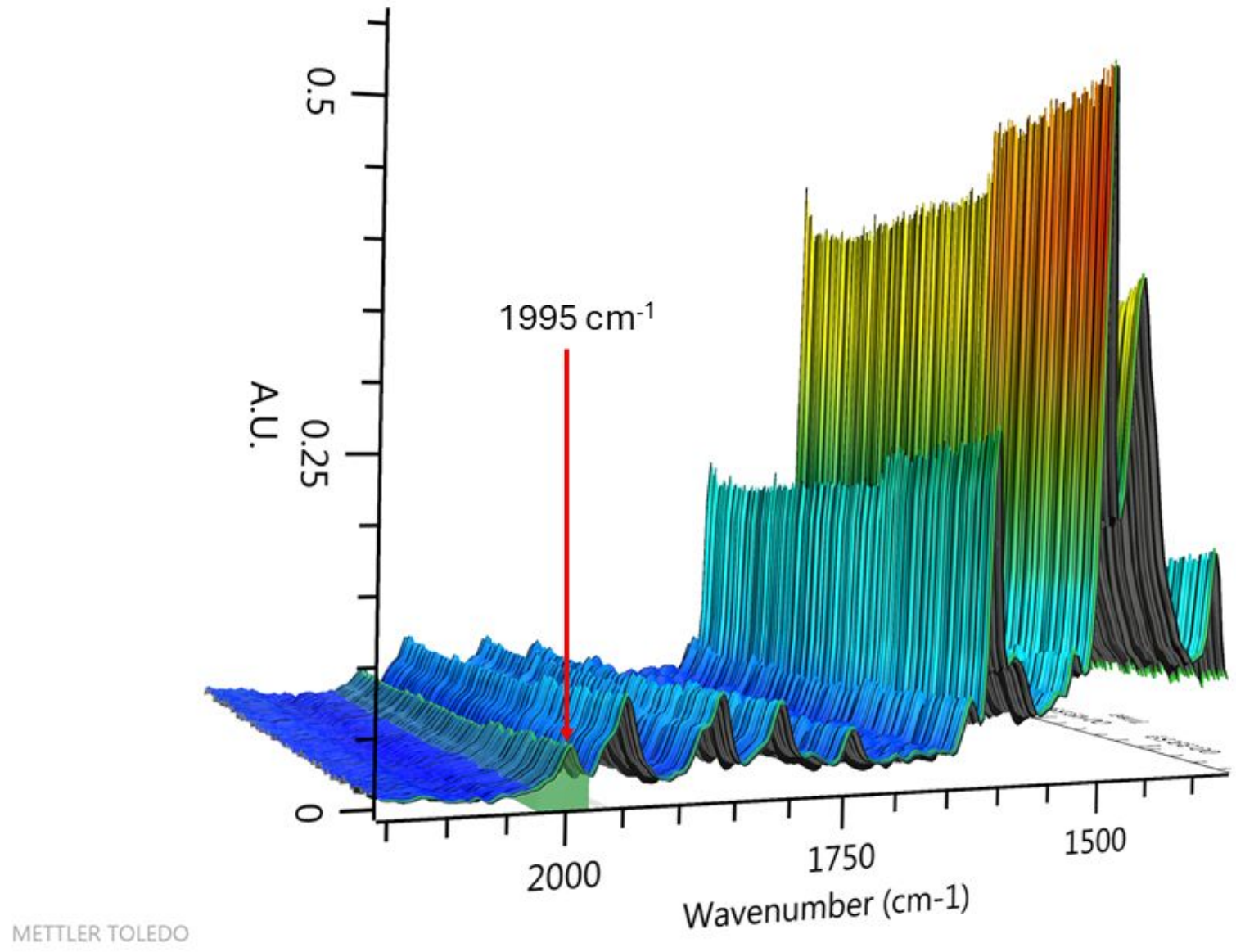

Figure S5. *In situ* IR spectra (React-IR, silicon probe) of the reaction between ^{t}Bu-M^{s}Fluind–Ga **1** with Ph_3PCN_2 at -60˚C.

2. Crystallographic Details

Data collection of all compounds was performed with a Bruker Venture D8 diffractometer at 100 K. For more details see the following Table S4 giving selected crystal and structural refinement data.

The structures of **2** - **4** were solved by direct methods using SHELXT 2018/2[7] and refined by full-matrix least-squares methods against F^2 by SHELXL-2018/3[8] employing shelXle (Revision 1449).[9] All non-hydrogen atoms were refined anisotropically while carbon-bonded hydrogen

atoms were placed on calculated positions isotropically. Disordered moieties were refined to two split occupancies using bond-length restraints and displacement-parameter restraints. The disorder models for specific groups (^{t}Bu, Cy) were introduced by the program DSR.[10]

For **2**, two ^{t}Bu units have been refined to split occupancies of 0.726/0.274 (C2A-C5A and C2B-C5B) and of 0.529/0.471 (C2C-C5C and C2D-C5D). Additionally, a cyclohexyl unit has been refined to split occupancies of 0.948/0.052 (C1_1-C6_1 and C1_2-C6_2). A benzene solvent molecule around a crystallographically imposed inversion center could not be refined reliably. As the unrefined electron density peaks were of very low intensity, the command SQUEEZE within PLATON[11] was applied. As result a solvent accessible void of 234 Å^3 with an electron count of 6 e/Å^3 was calculated. This very low electron count indicates that this void is sparingly occupied by benzene with a theoretical electron count of 42 e/Å^3 per benzene molecule. A non-disordered benzene molecule would occupy a space of 120 Å^3, a disordered benzene molecule a space of 240 Å^3. This agrees with the SQUEEZE calculated volume of 234 Å^3. Considering the calculated electron count in the void of 6 e/Å^3, the theoretical electron count of 42 e/Å^3 and Z = 4, this results in 1/28 benzene content per molecule.

For **3**, a ^{t}Bu unit has been refined to split occupancies of 0.642/0.195/0.163 (C2_1-C5_1, C2_3-C5_3, C2_2-C5_2) using the SUMP command. A benzene solvent molecule was found around a crystallographically imposed inversion center with an occupancy of 0.15.

All graphics of the molecular solid-state structures were created using OLEX2 (version 1.5).[12]

CCDC 2514151 (**2**), 2514152 (**3**) and 2514153 (**4**) contain supplementary crystallographic data. These data can be obtained free of charge from The Cambridge Crystallographic Data Centre via www.ccdc.cam.ac.uk/data_request/cif.

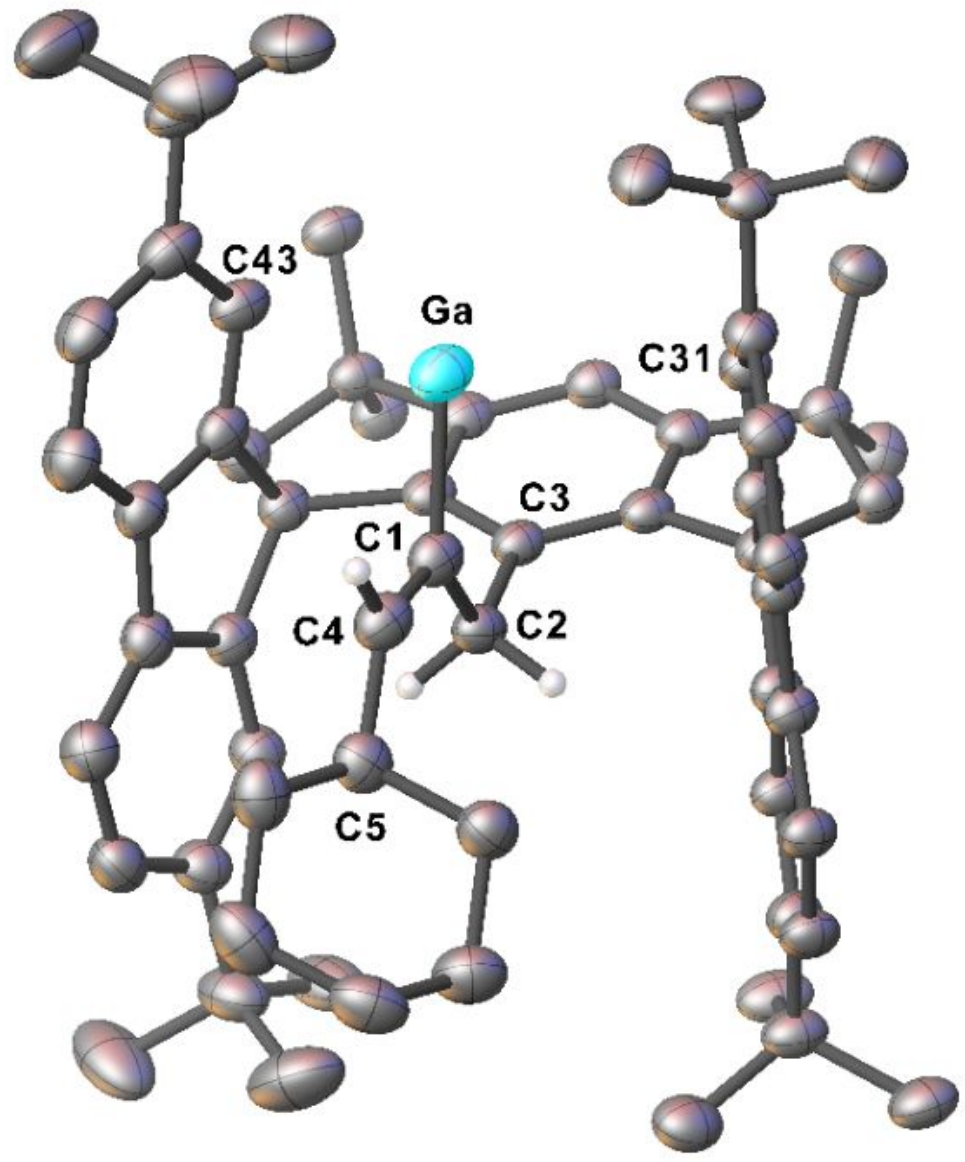

Figure S6. Molecular solid-state structure of **2** (ellipsoids represent 50% probability). Selected bond lengths / distances (Å): C1–Ga 2.007(3), C1–C2 1.497(4), C2–C3 1.533(4), C1–C4 1.345(5), C4–C5 1.505(5), Ga···Ga 9.735, Ga···C43 3.159(3), Ga···C31 3.224(3). Second molecule of **2** in asymmetric unit (with ^{t}Bu and cyclohexyl disorder) and hydrogen atoms are omitted for clarity.

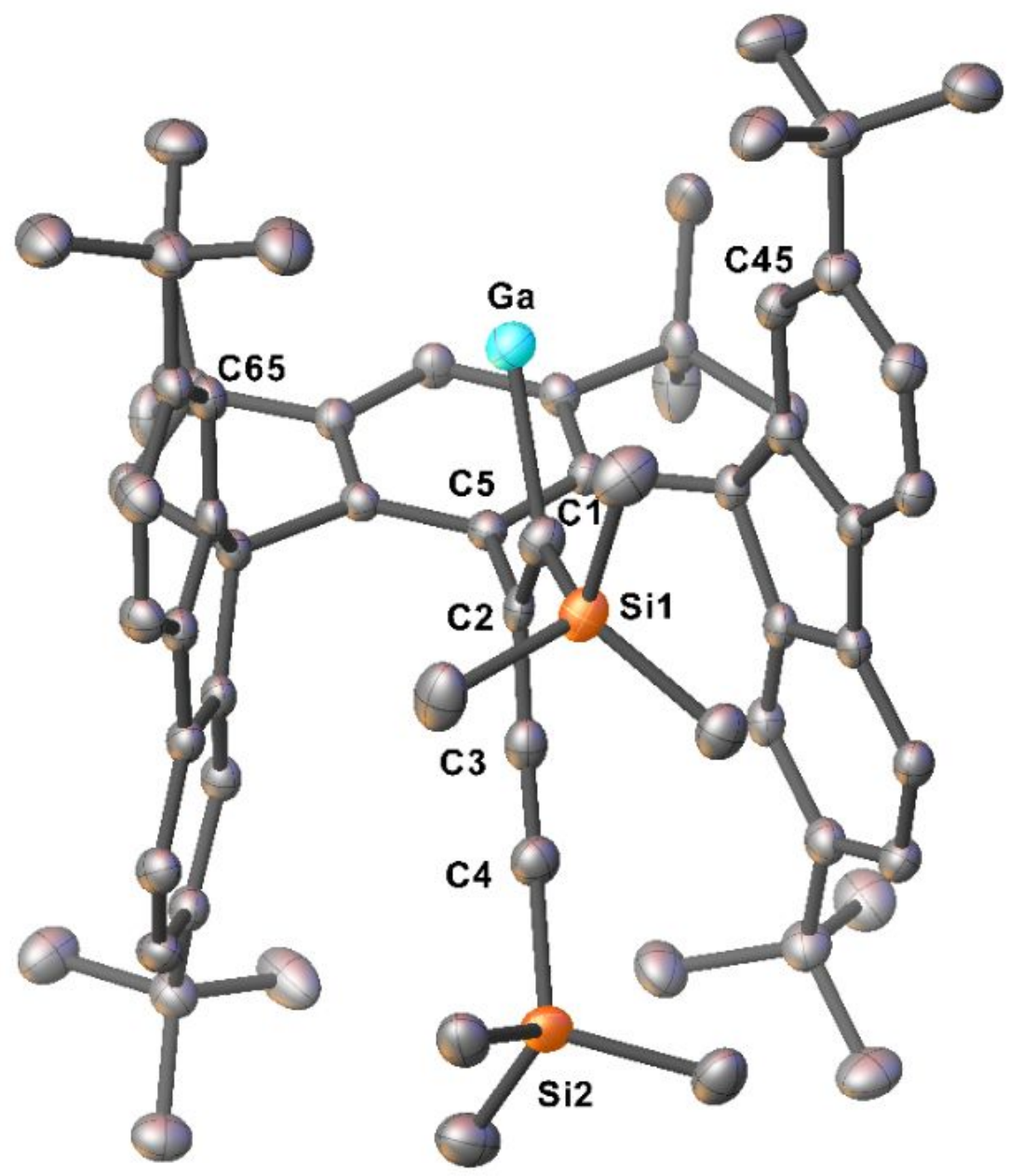

Figure S7. Molecular solid-state structure of **3** (ellipsoids represent 50% probability). Selected bond lengths / distances (Å) and angles (°): C1–Ga 2.072(2), C1–C2 1.359(3), C2–C3 1.423(3), C3–C4 1.215(3), C2–C5 1.543(2), C1–Si1 1.8762(19), C4–Si2 1.833(2), C2–C3–C4 171.8(2), C3–C4–Si2 167.77(19), Ga···Ga 9.255, Ga···C45 2.9553(18), Ga···C65 3.2005(18). Second molecule of **3** in asymmetric unit with disordered ^{t}Bu group, two benzene solvent molecules and hydrogen atoms are omitted for clarity.

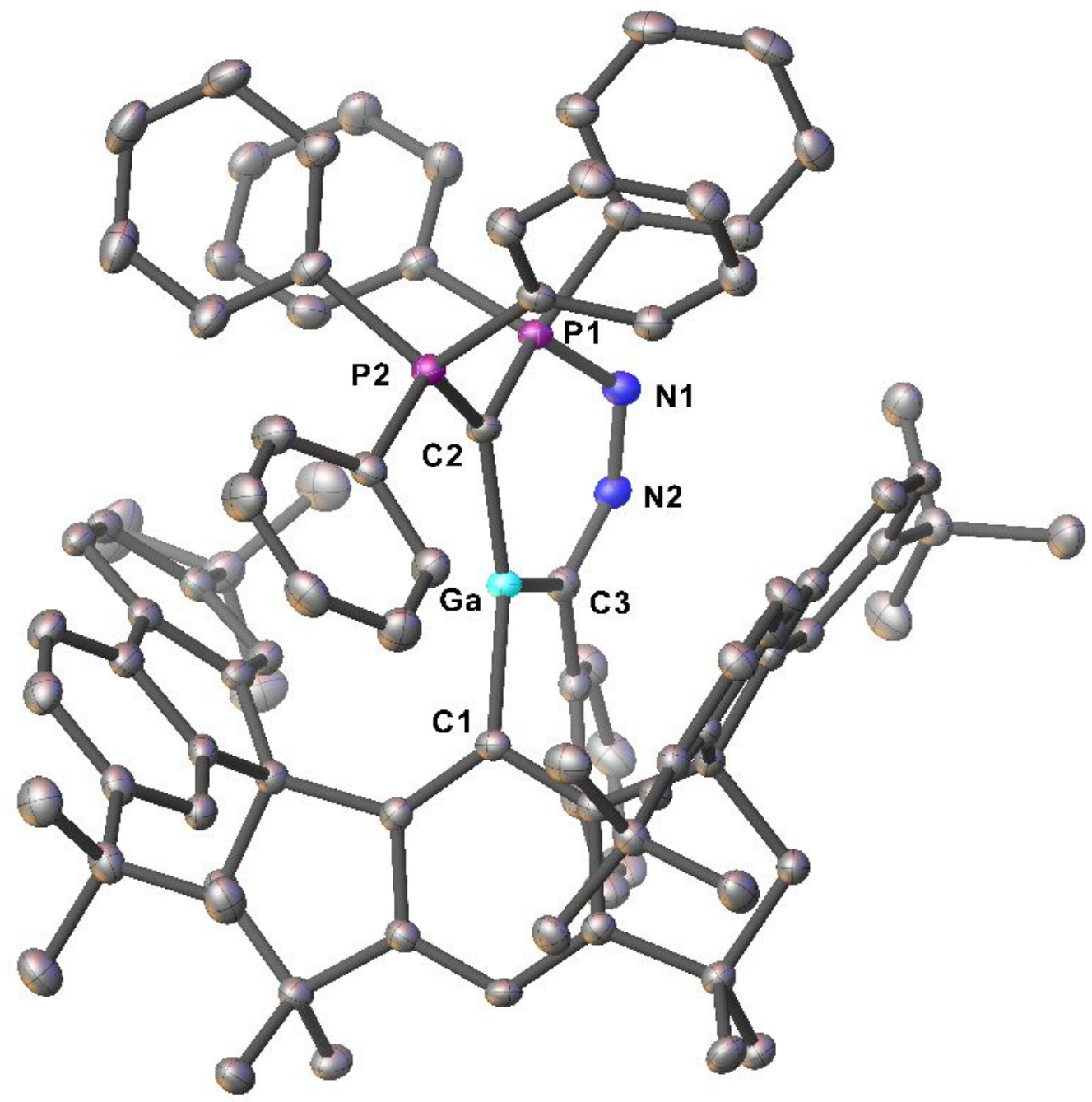

Figure S8. Molecular solid-state structure of **4** (ellipsoids represent 50% probability). Selected bond lengths (Å) and angles (˚): C1–Ga 1.9988(18), Ga–C2 1.9278(18), Ga–C3 1.9911(19), C2–P1 1.7743(18), C2–P2 1.7204(19), P1–N1 1.6206(16), N1–N2 1.352(2), N2–C3 1.313(2), Ga–C2–P1 107.10(9), P2–C2–P1 118.98(10), P2–C2–Ga 133.92(10), C2–P1–N1 117.75(8), P1–N1–N2 127.87(13), N1–N2–C3 124.93(16), N2–C3–Ga 120.84(14), C2–Ga–C3 110.54(8). Hydrogen atoms are omitted for clarity.

Table S4. Crystal and structural refinement data of **2** - **4**.

	**2**	**3**	**4**
Empirical formula	$C_{65}H_{79}Ga$ + [1/28 C_6H_6][1]	$C_{69.45}H_{86.45}GaSi_2$	$C_{94}H_{95}GaN_2P_2$
Formula mass (g·mol^{-1})	930.00	1047.15	1384.37
Crystal system	triclinic	triclinic	monoclinic
Space group	*P-1*	*P-1*	*C2/c*
a (Å)	15.0918(5)	14.7900(3)	22.1409(4)
b (Å)	15.1171(5)	18.3731(4)	14.1224(2)
c (Å)	28.0507(10)	23.7850(5)	48.3364(8)
α (°)	79.114(2)	84.056(1)	90
β (°)	79.848(2)	73.449(1)	100.8800(10)
γ (°)	61.753(2)	88.467(1)	90
V (Å^3)	5508.1(3)	6162.2(2)	14842.3(4)
Z	4	4	8
D_{calc} (Mg·m^{-3})	1.121	1.129	1.239
Wavelength (Å)	1.54178	1.54178	1.54178
Absorption coefficient (mm^{-1})	0.950	1.261	1.283
F(000)	2000	2249	5872
Reflection collected	118077	144163	127792
Reflection unique / R_{int}[a)]	20730 / 0.0803	23330 / 0.0469	14610 / 0.0738
Goodness-of-fit on F^2[b)]	1.039	1.034	1.051
Data / restrains / parameters	20730 / 687 / 1357	23330 / 556 / 1476	14610 / 0 / 908
θ range for data collection (°)	3.225 to 70.29	1.947 to 70.502	3.725 to 74.493
Limiting indices	$-18 \le h \le 18$, $-18 \le k \le 17$, $-34 \le l \le 34$	$-18 \le h \le 18$, $-22 \le k \le 22$, $-29 \le l \le 29$	$-27 \le h \le 26$, $-17 \le k \le 17$, $-57 \le l \le 59$
Final R indices [$I > 2\sigma(I)$][c)]	R_1 = 0.0762, wR_2 = 0.2133	R_1 = 0.0453, wR_2 = 0.1204	R_1 = 0.0392, wR_2 = 0.0945
R indices (all data)[c)]	R_1 = 0.0901, wR_2 = 0.2279	R_1 = 0.0517, wR_2 = 0.1259	R_1 = 0.0513, wR_2 = 0.1018
Largest diff. peak/hole (e·Å^{-3})	0.77/-1.61	1.07/-0.70	0.45/-0.47

[a] $R_{int} = \sum|F_0^2 - F_0^2(\mathrm{mean})|/\sum F_0^2$, where F_0^2 (mean) is the average intensity of symmetry equivalent diffractions. [b] $S = [\sum w(F_0^2 - F_0^2)^2]/(n\text{-}p)^{1/2}$, where n = number of reflections, p = number of parameters. [c] $R = [\sum(\|F_0| - |F_0|)/\sum|F_0|)$; $R[\sum(w(F_0^2 - F_0^2))^2/\sum(wF_0^4)]^{1/2}$ [1] The dataset was treated with SQUEEZE. A reasonable fit of the calculated electron count and void volume is 1/28 molecule of benzene, which has been used for crystallization.

3. Spectra

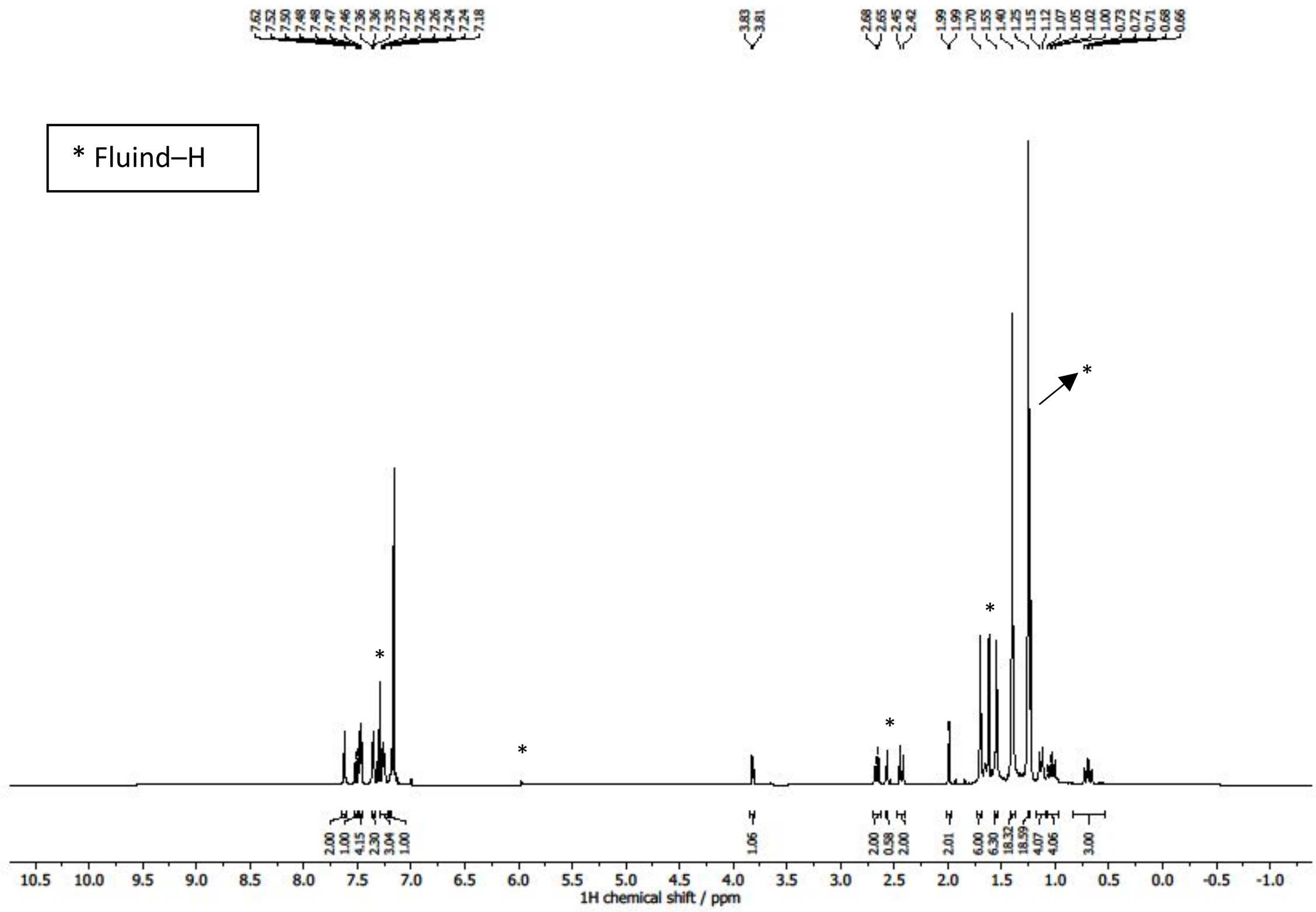

Figure S9. ^{1}H NMR spectrum (500 MHz, C_6D_6, 298 K) of **2**. Signals from proteo-Ligand Fluind–H marked with *.

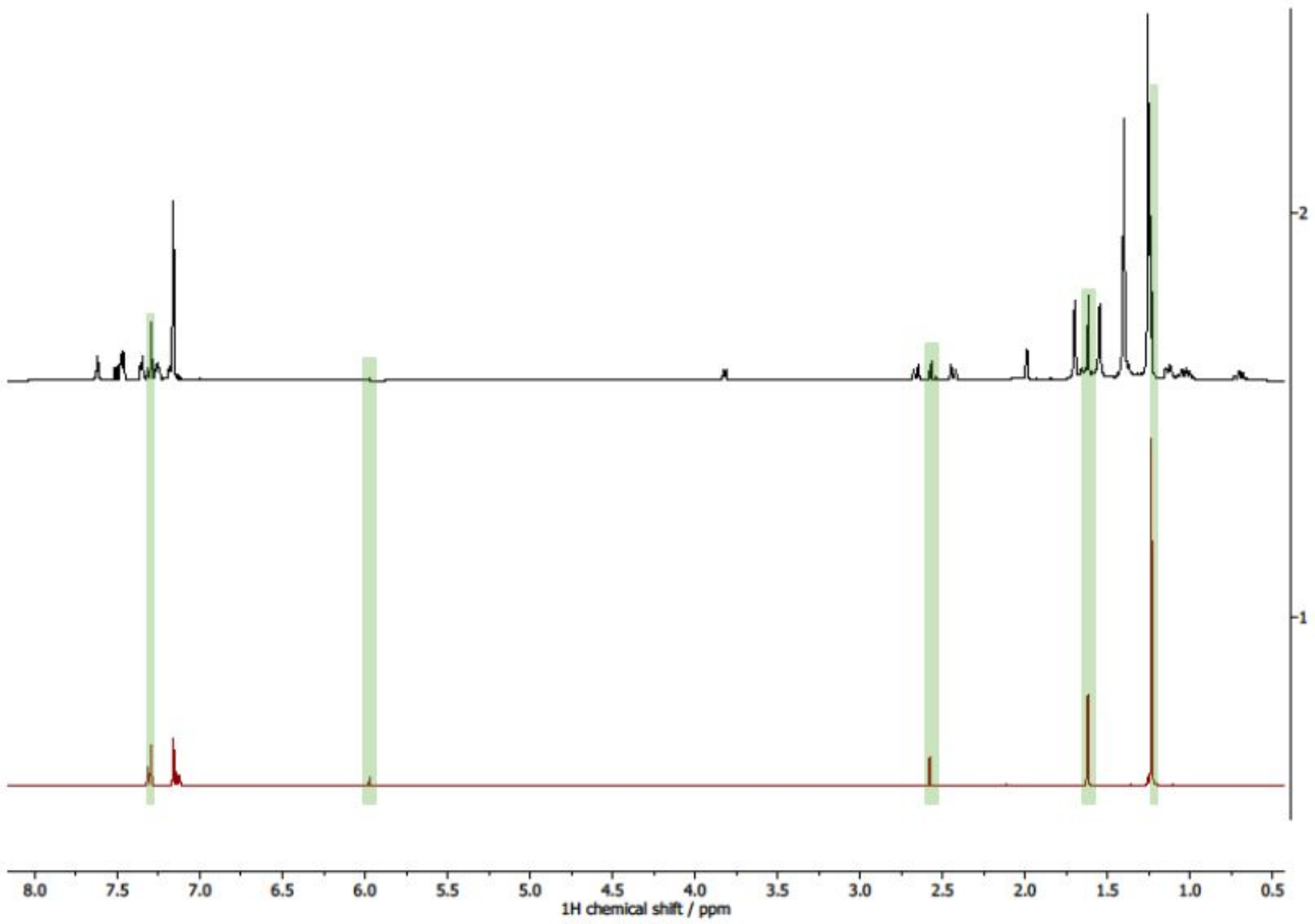

Figure S10. ^{1}H NMR spectrum (500 MHz, C_6D_6, 298 K) of **2** (top, black) and proteo-Ligand Fluind–H (bottom, red) showing contamination.

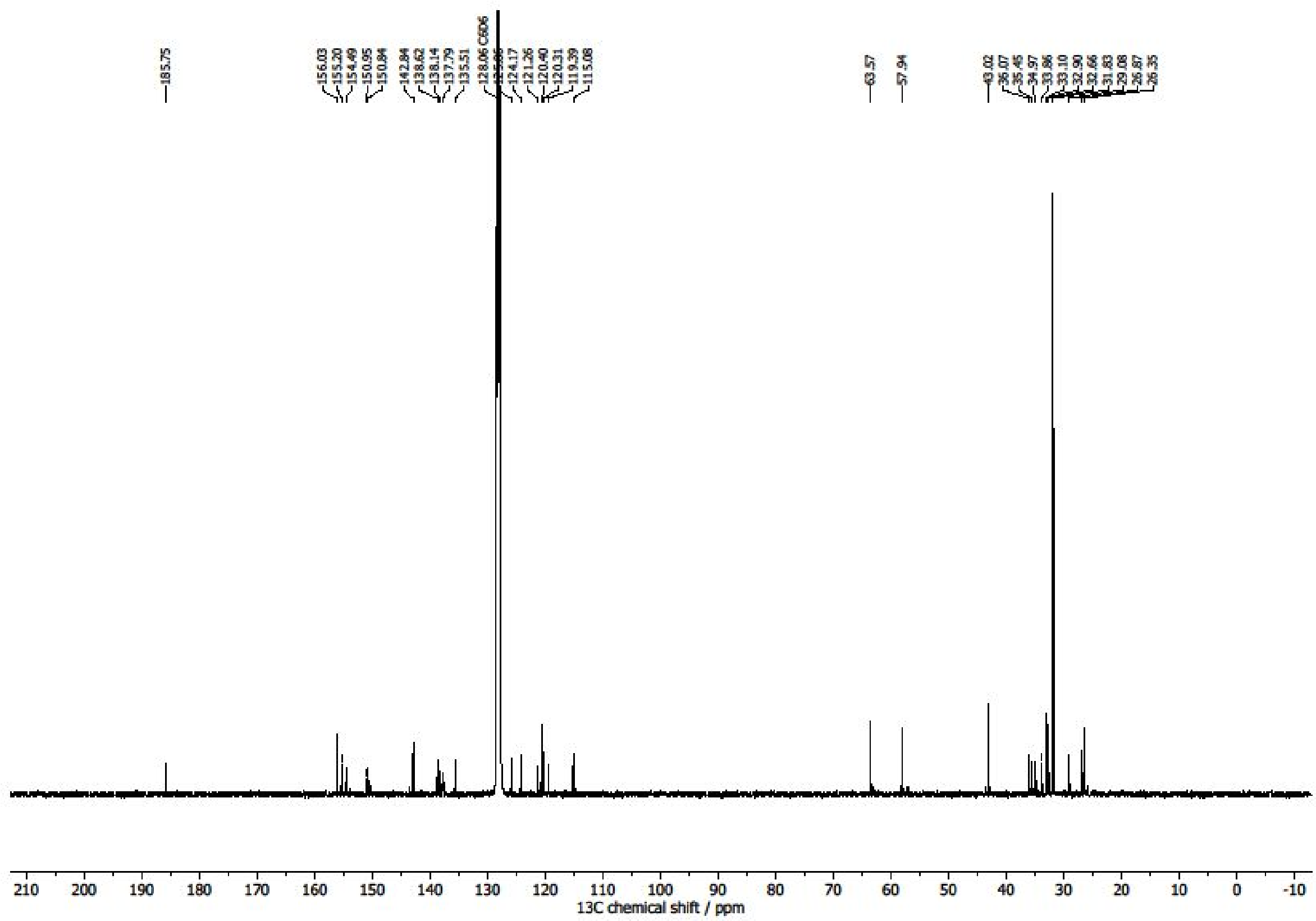

Figure S11. $^{13}C\{^{1}H\}$ NMR spectrum (121 MHz, C_6D_6, 298 K) of **2**.

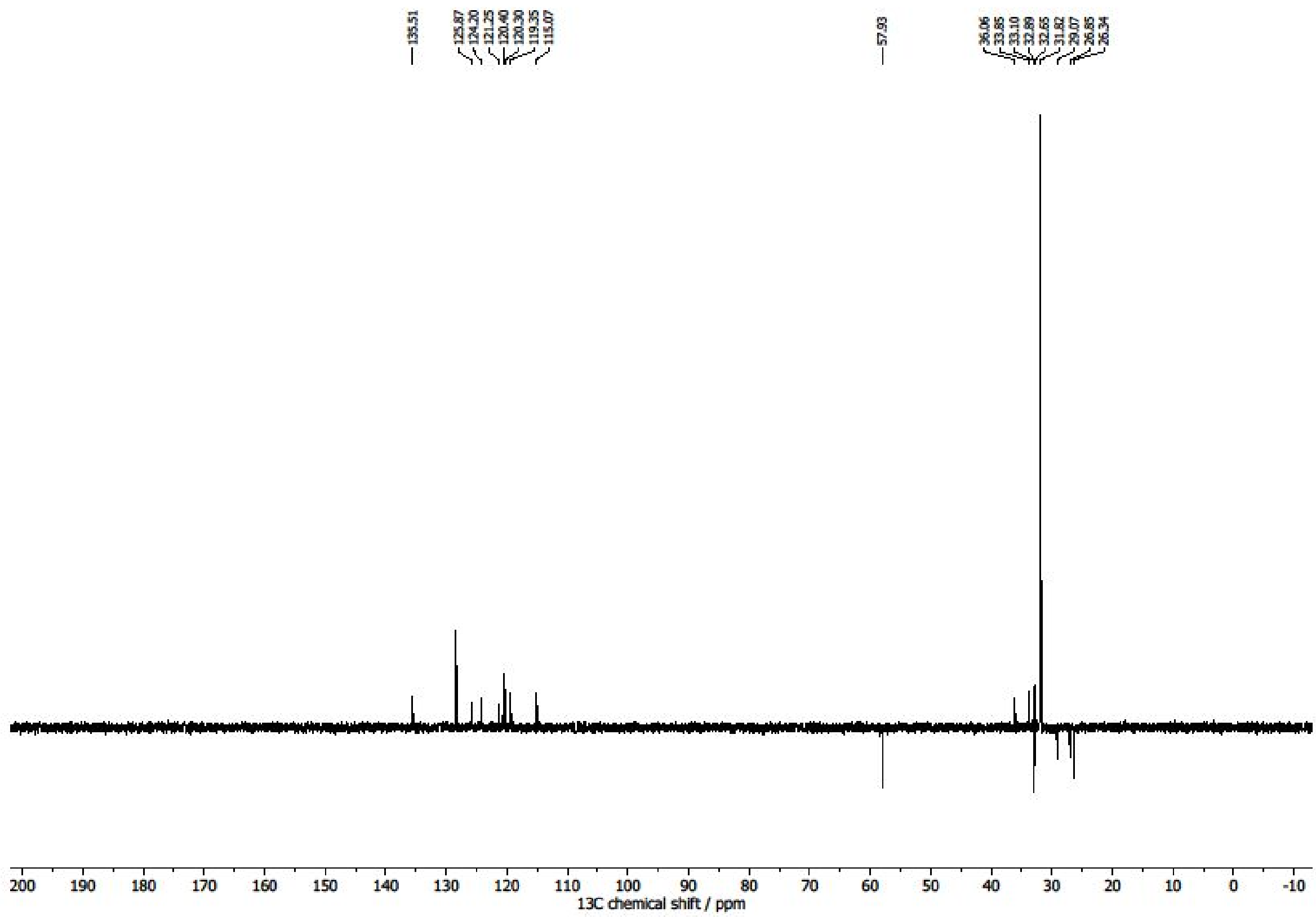

Figure S12. $^{13}C\{^{1}H\}$ DEPT135 NMR spectrum (121 MHz, C_6D_6, 298 K) of **2**.

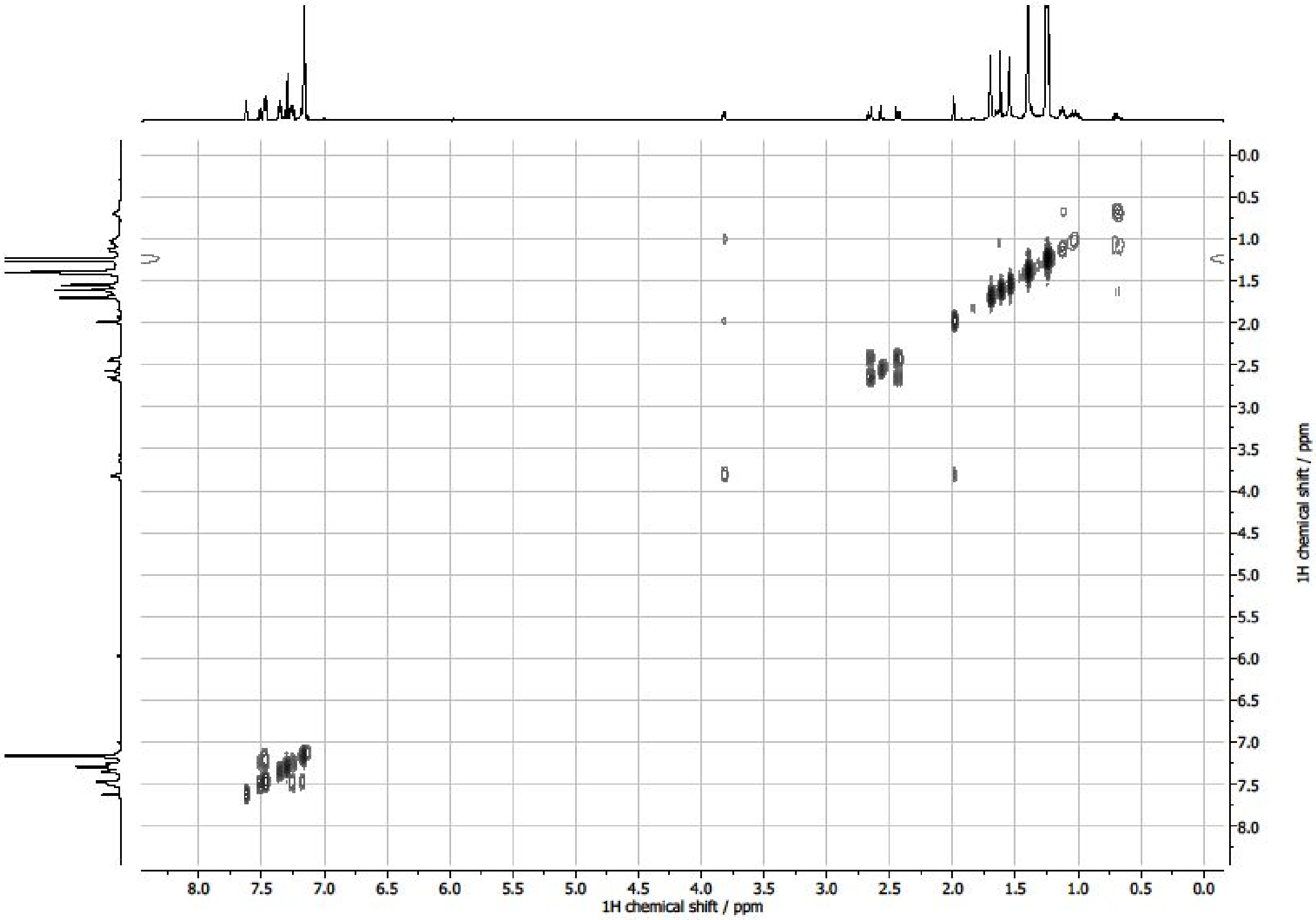

Figure S13. COSY NMR spectrum (500 MHz, C_6D_6, 298 K) of **2**.

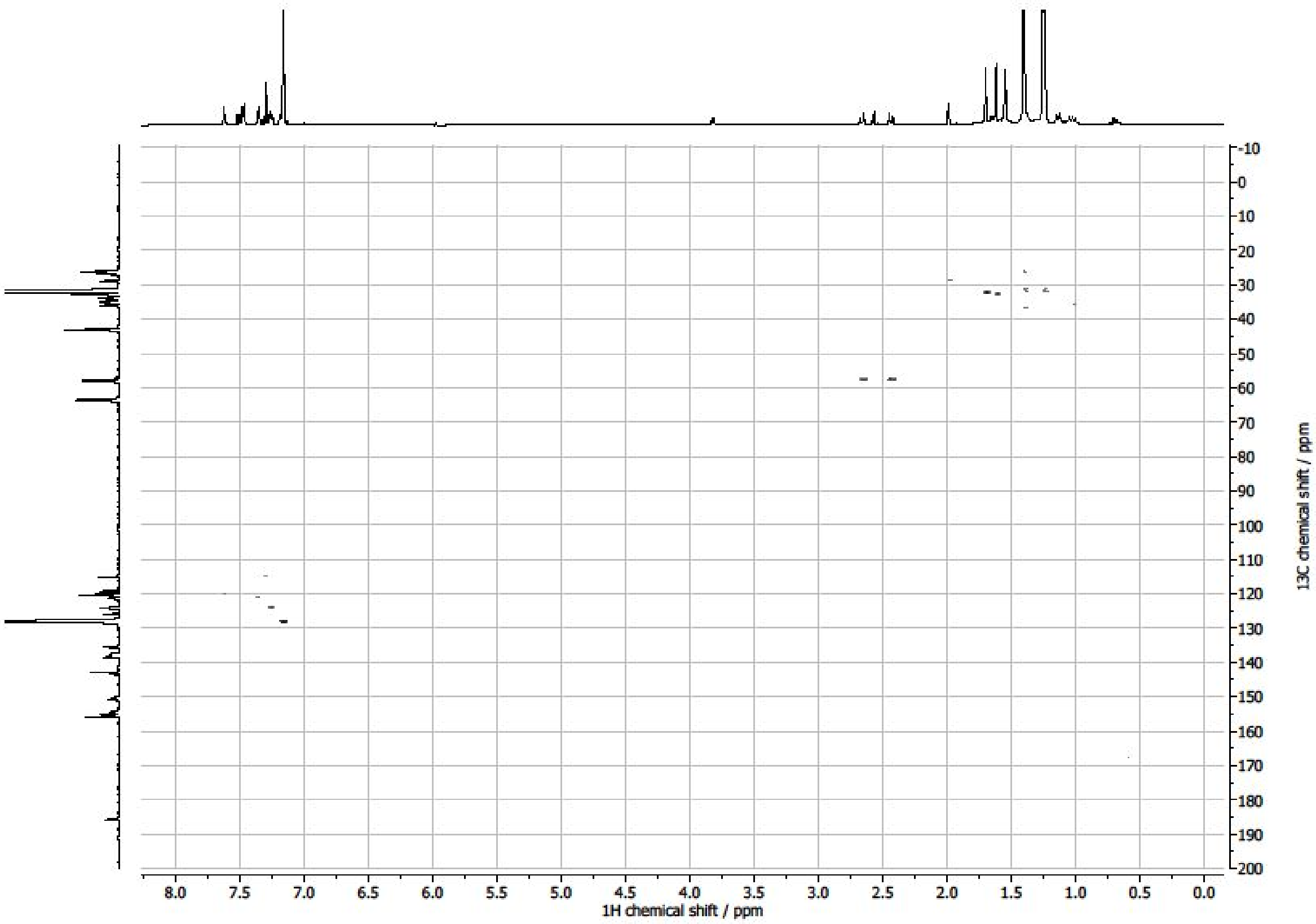

Figure S14. ^{1}H-^{13}C HSQC spectrum (C_6D_6, 298 K) of **2**.

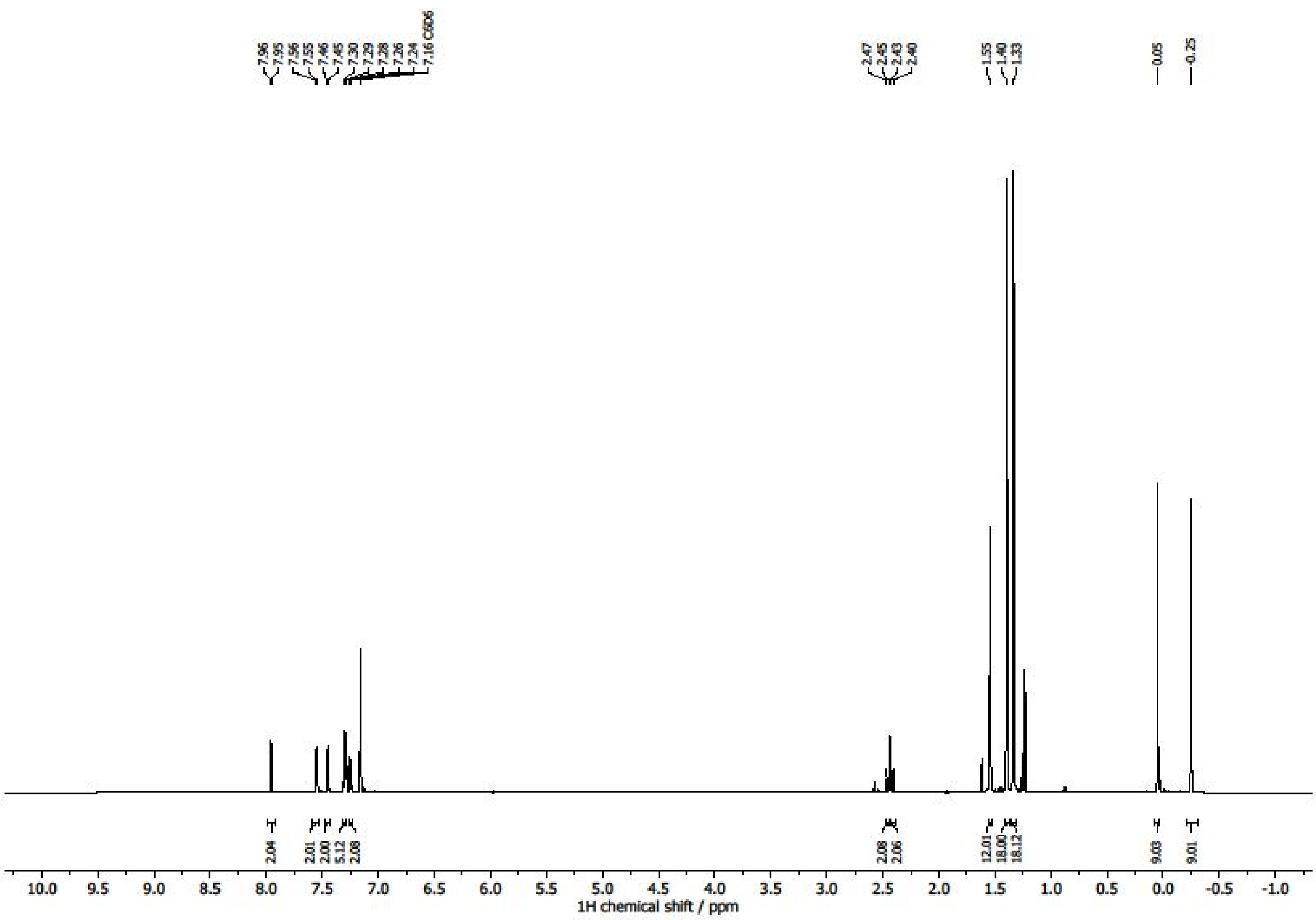

Figure S15. ^{1}H NMR spectrum (500 MHz, C_6D_6, 298 K) of 3.

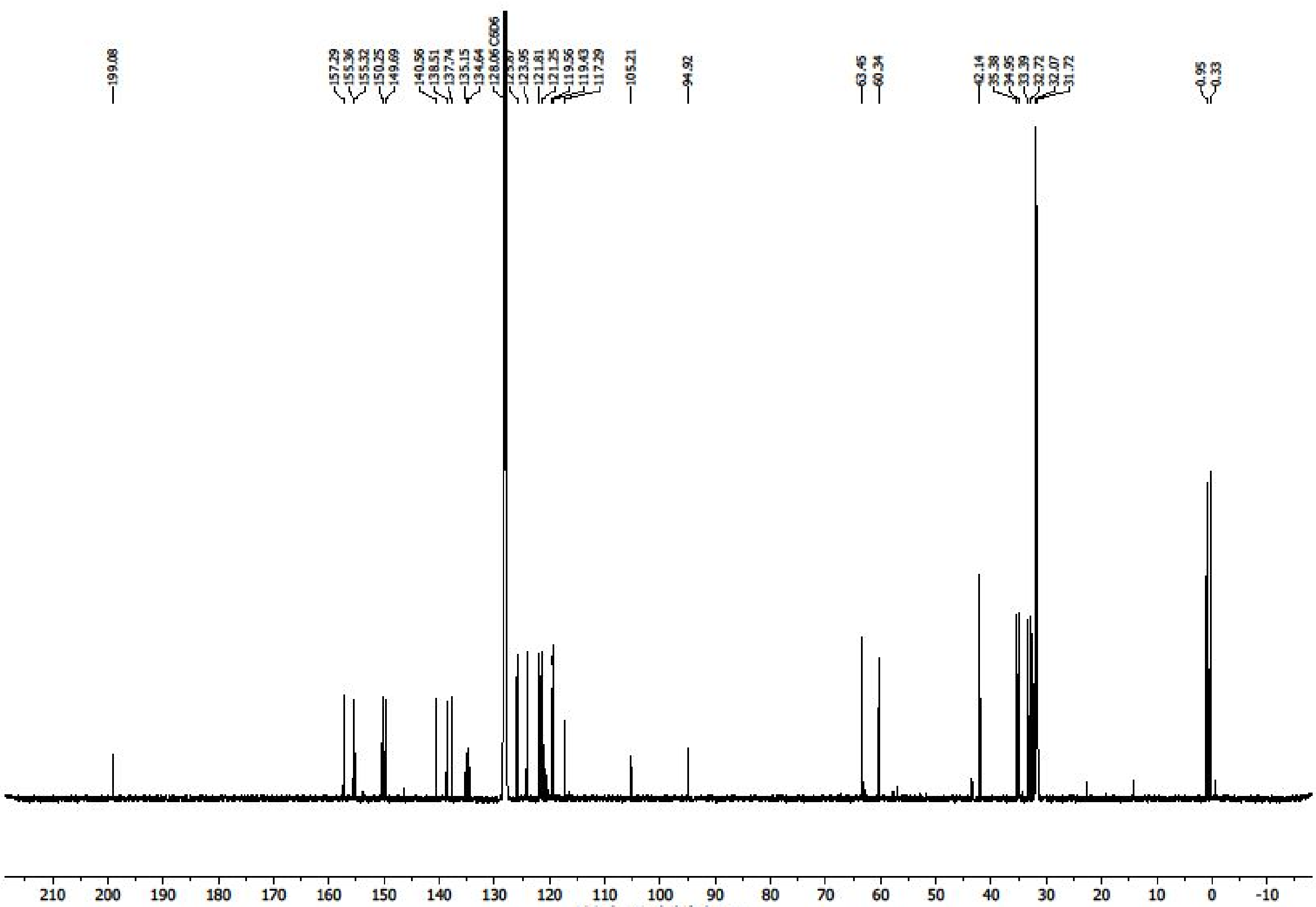

Figure S16. $^{13}C\{^{1}H\}$ NMR spectrum (121 MHz, C_6D_6, 298 K) of 3.

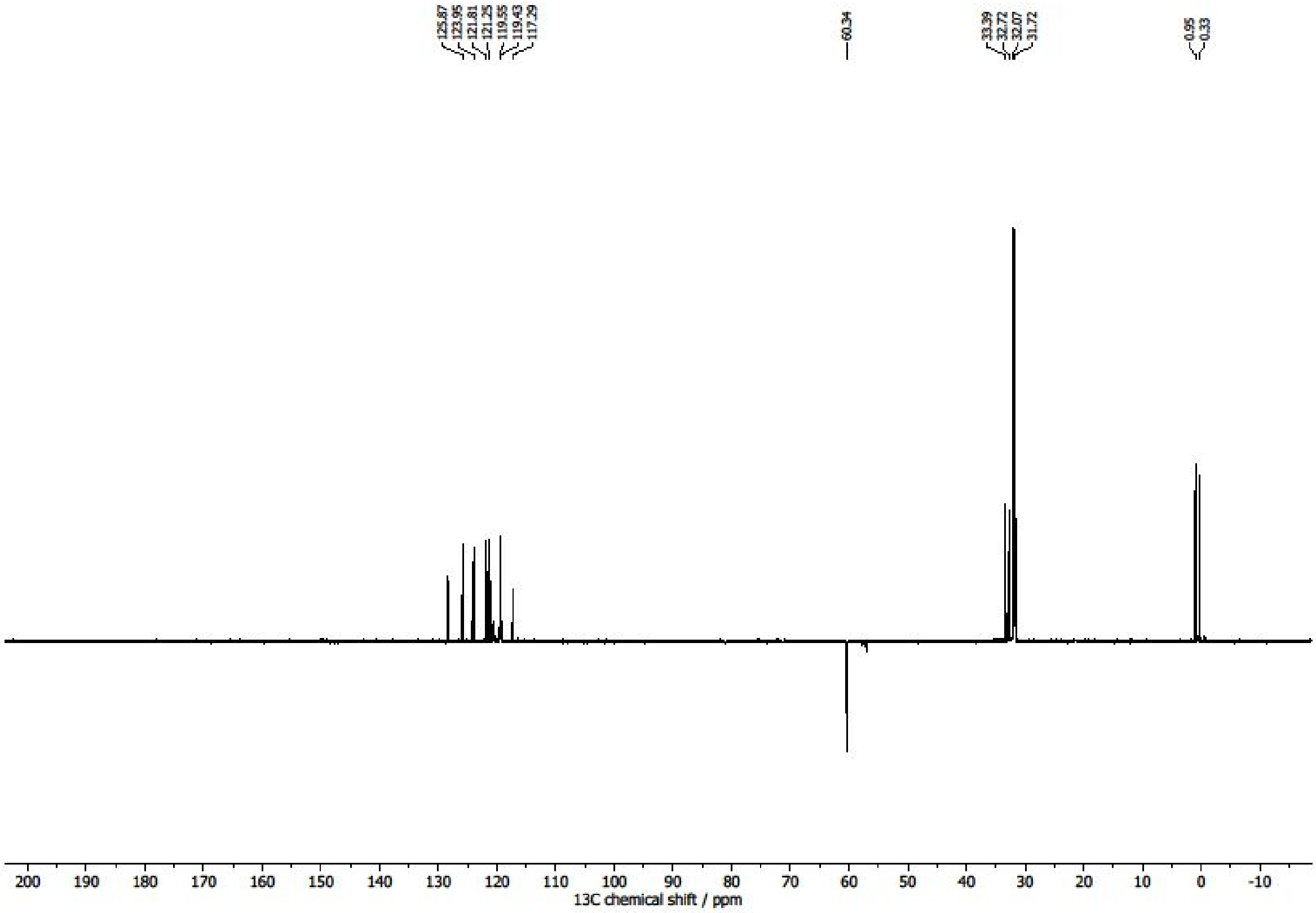

Figure S17. $^{13}C\{^1H\}$ DEPT135 NMR spectrum (121 MHz, C_6D_6, 298 K) of **3**.

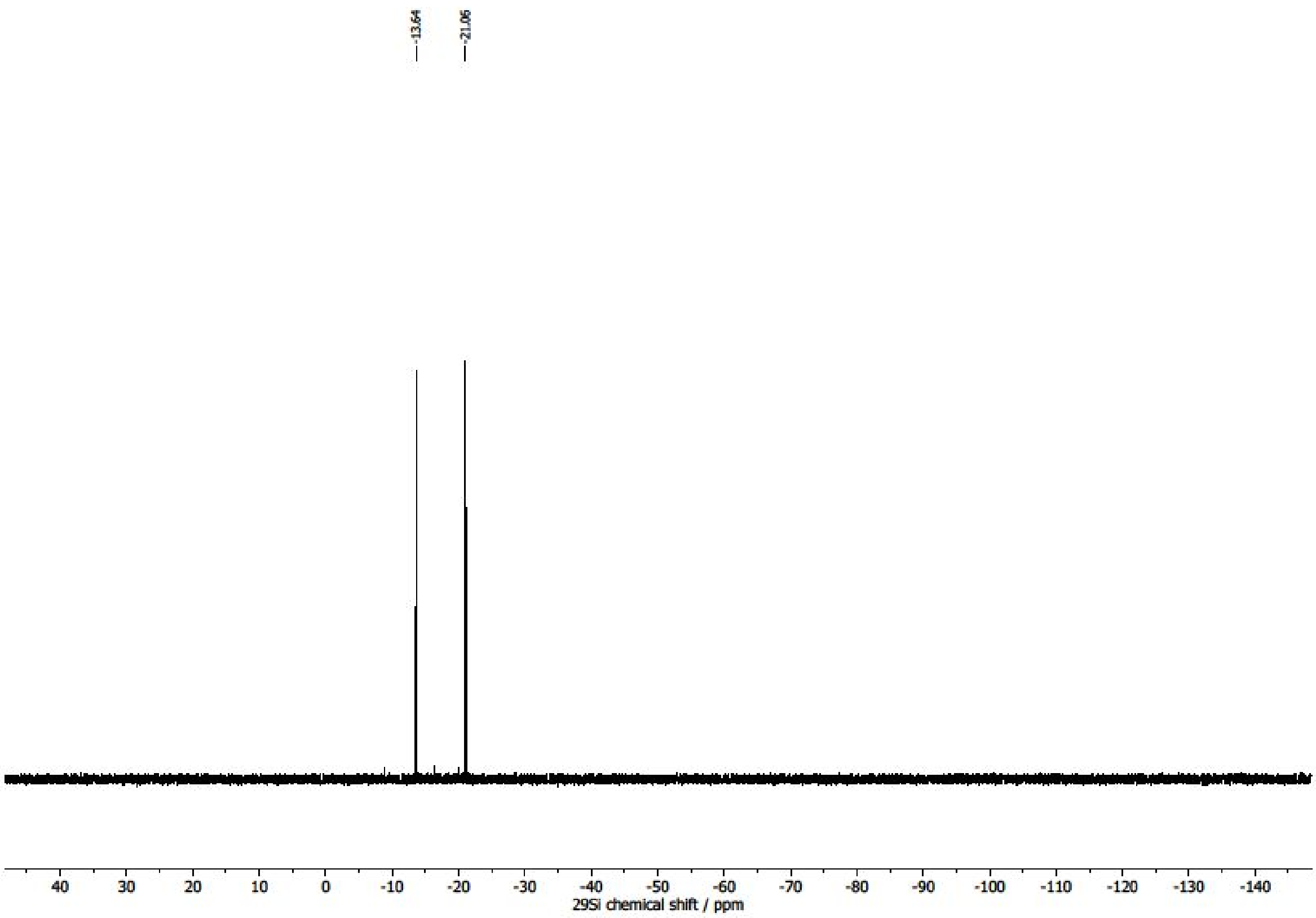

Figure S18. $^{29}Si\{^1H\}$ DEPT135 NMR spectrum (99 MHz, C_6D_6, 298 K) of **3**.

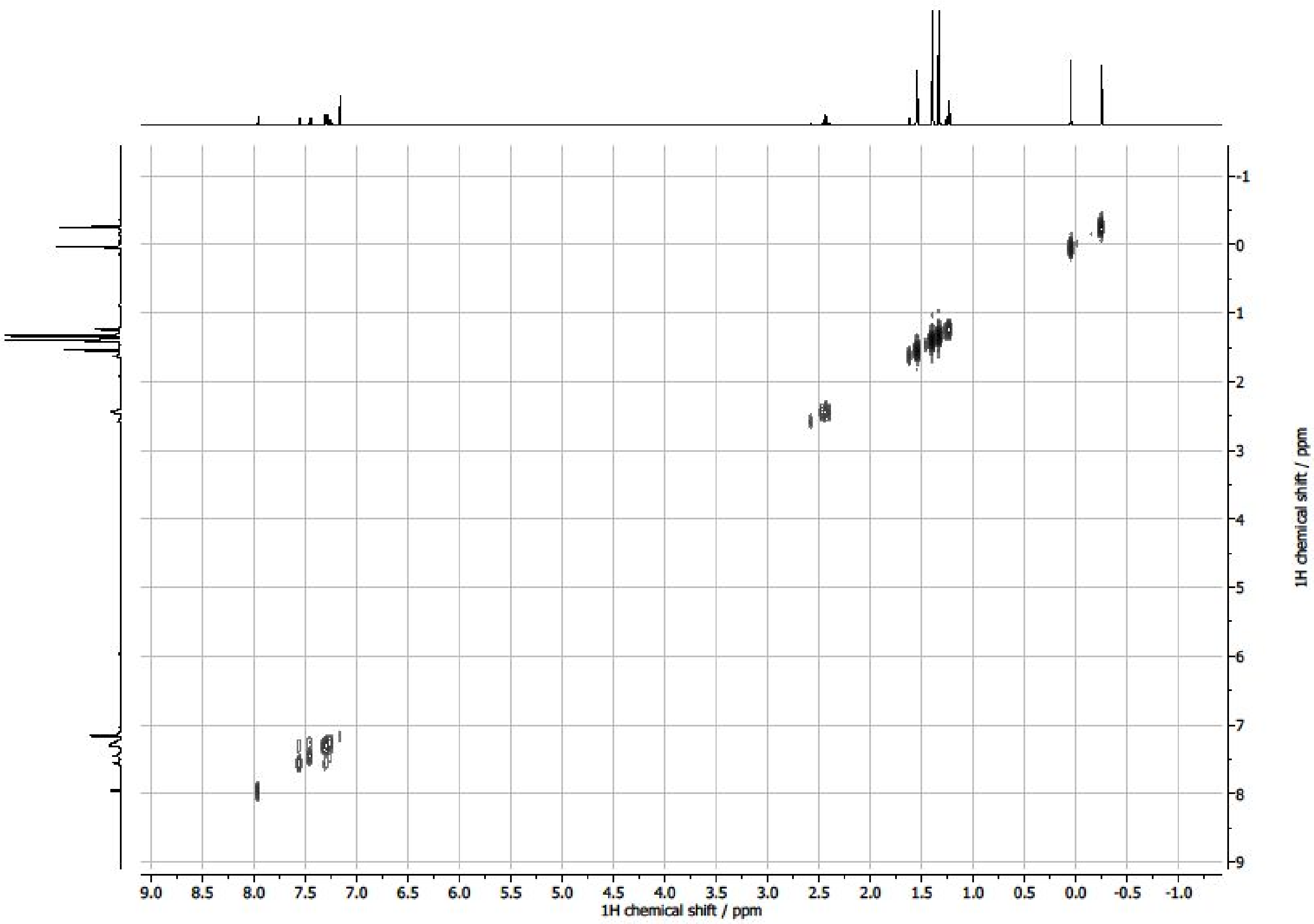

Figure S19. COSY NMR spectrum (500 MHz, C_6D_6, 298 K) of **3**.

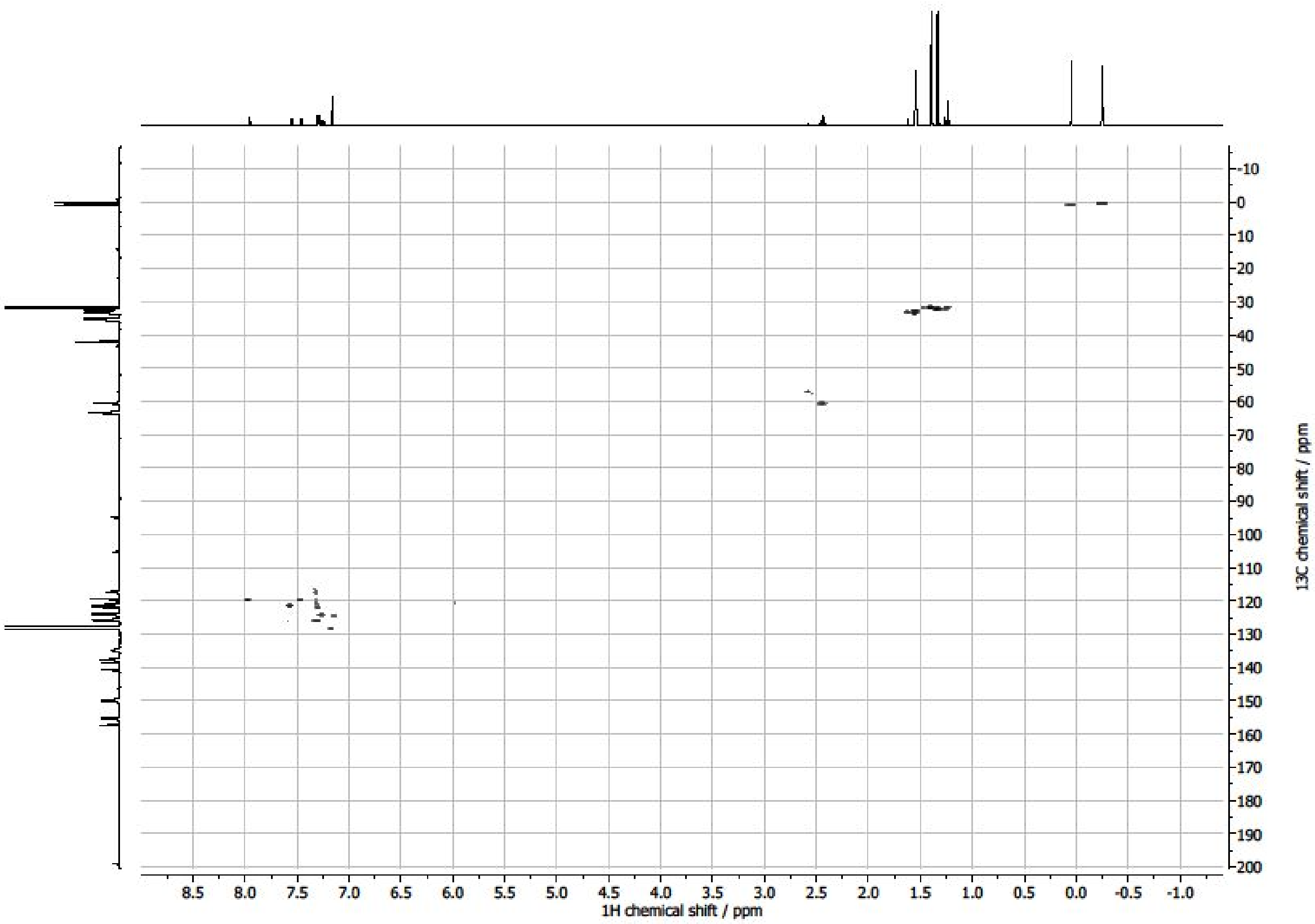

Figure S20. ^{1}H-^{13}C HSQC spectrum (C_6D_6, 298 K) of **3**.

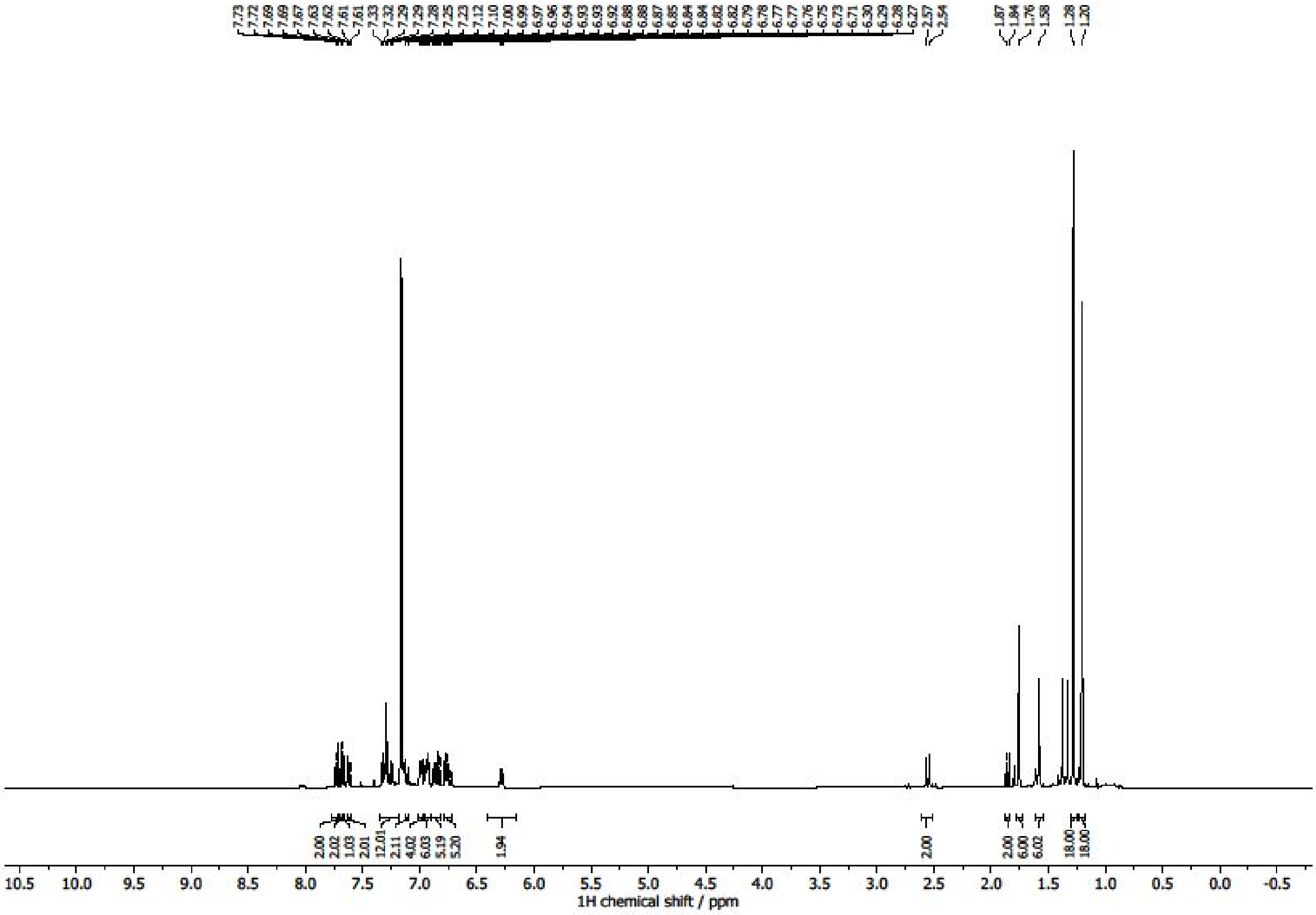

Figure S21. ^{1}H NMR spectrum (500 MHz, C_6D_6, 298 K) of **4**.

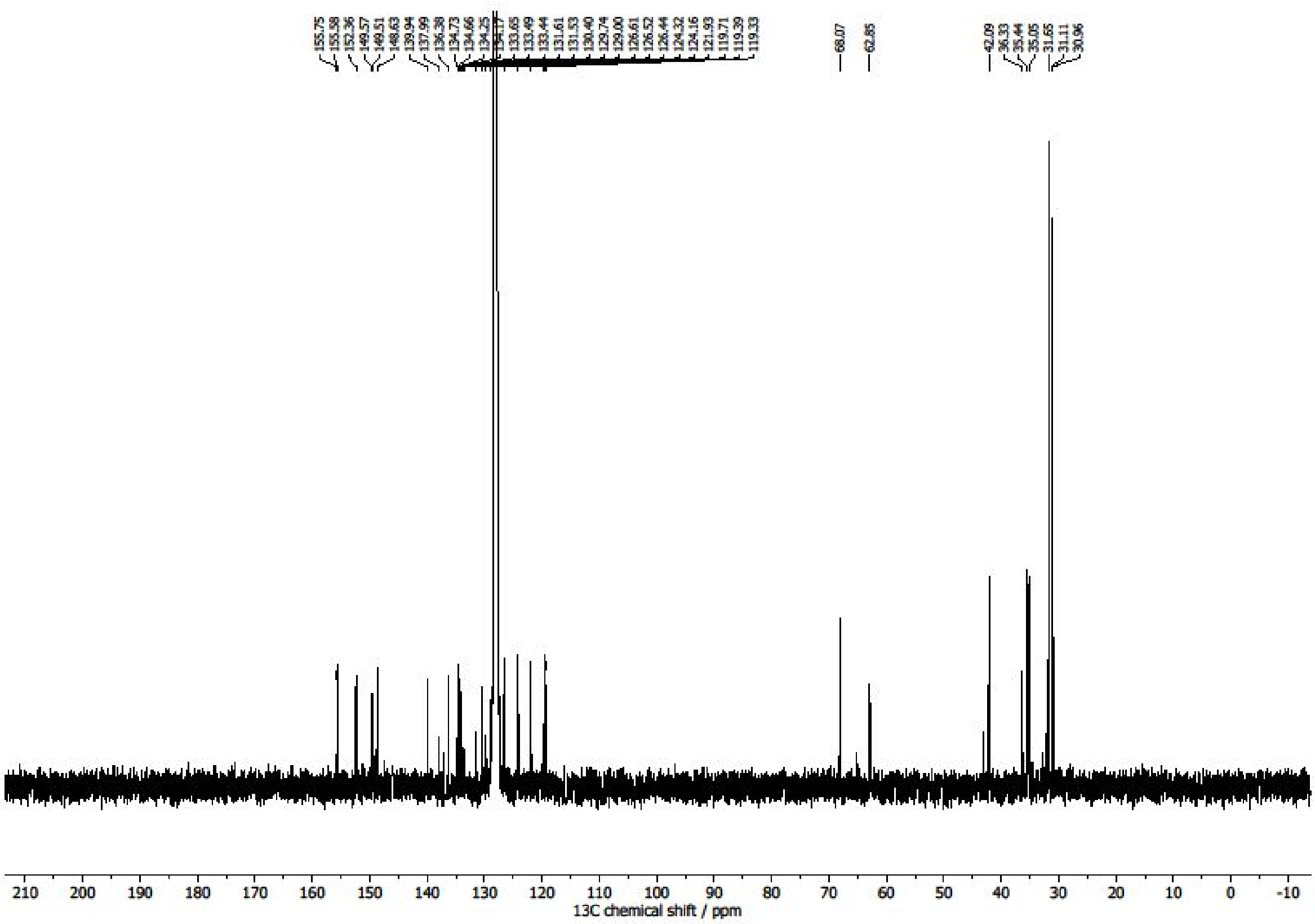

Figure S22. $^{13}C\{^{1}H\}$ NMR spectrum (121 MHz, C_6D_6, 298 K) of **4**.

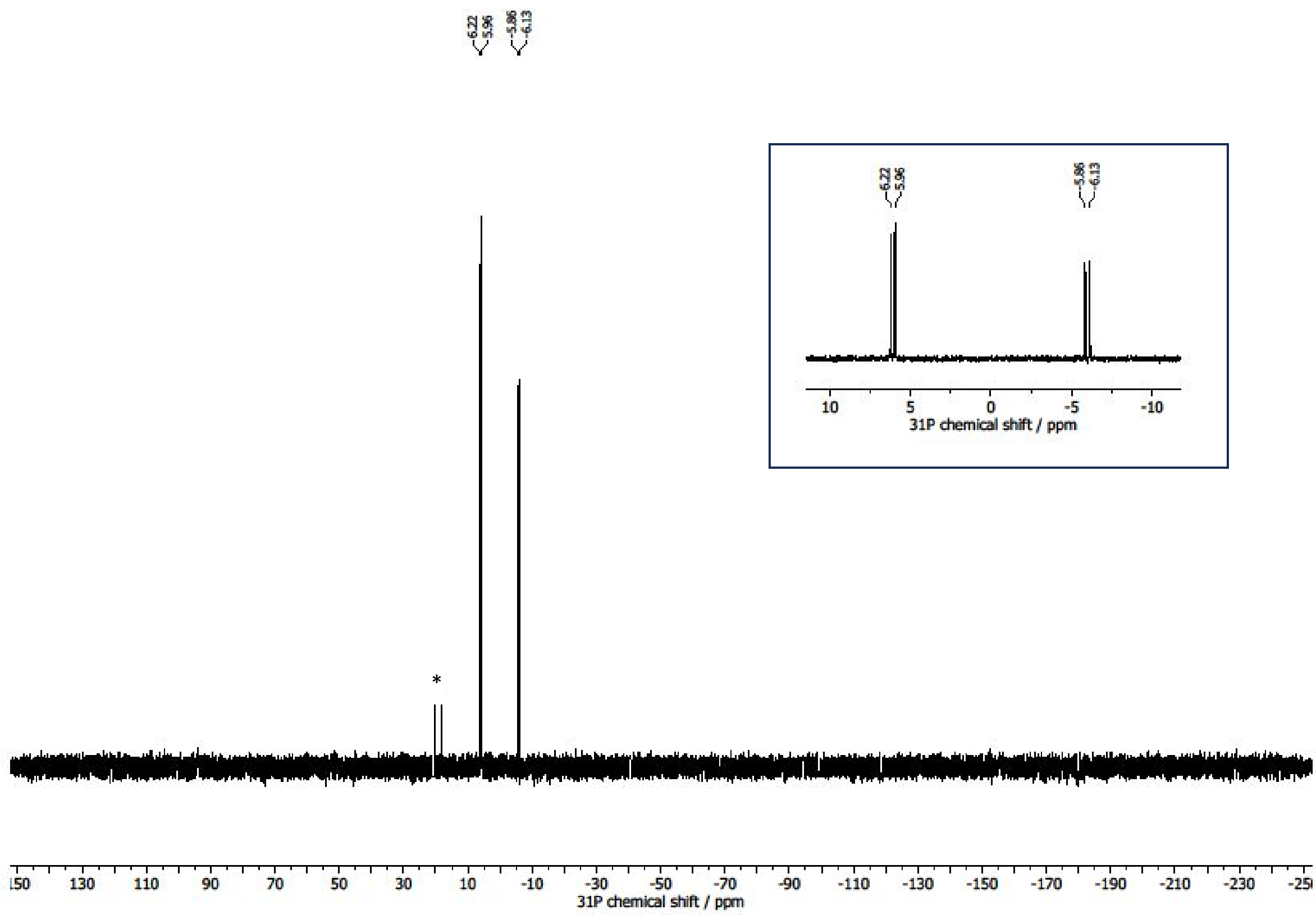

Figure S23. $^{31}P\{^{1}H\}$ NMR spectrum (203 MHz, C_6D_6, 298 K) of **4**. Side product marked with *.

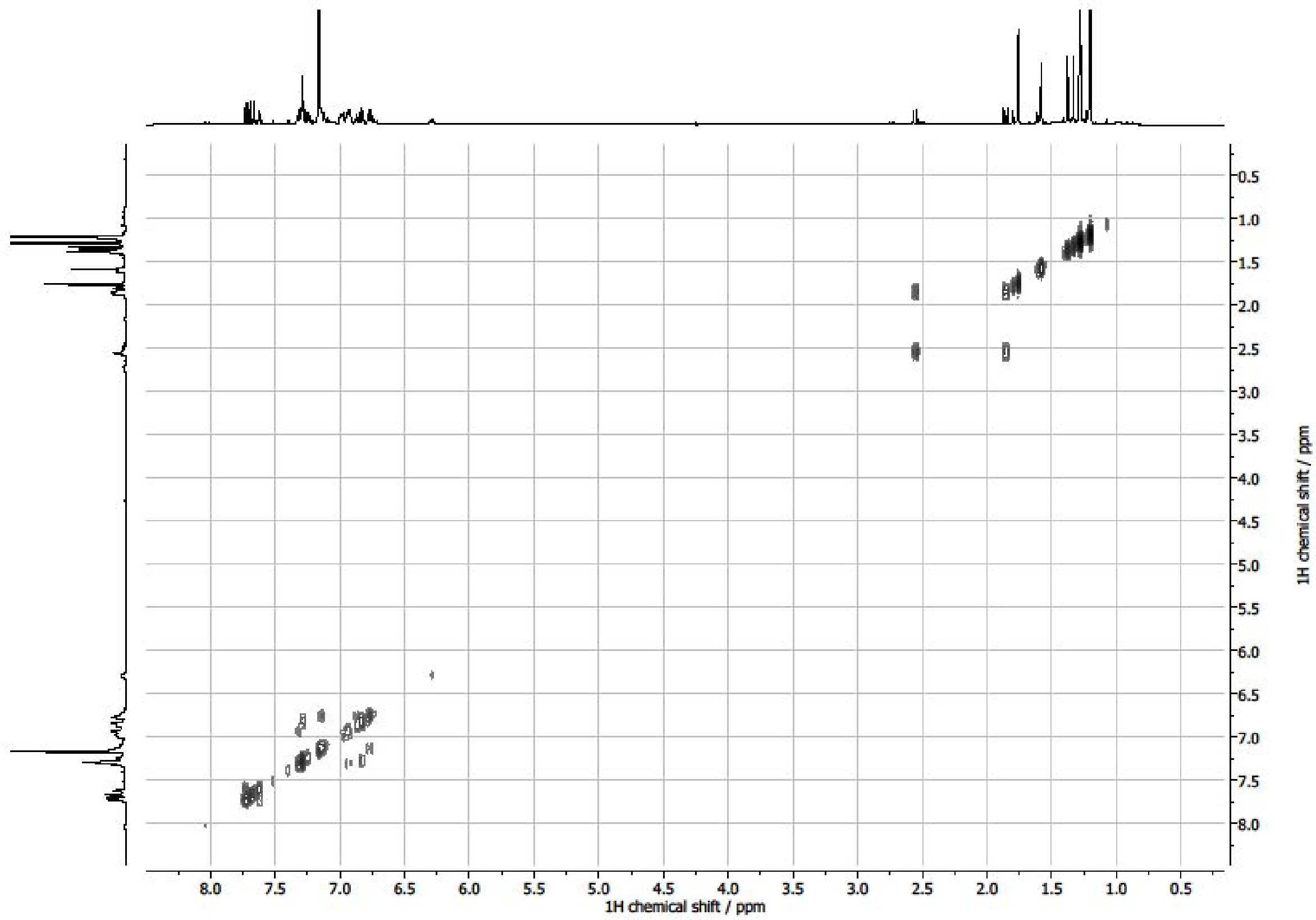

Figure S24. COSY NMR spectrum (500 MHz, C_6D_6, 298 K) of **4**.

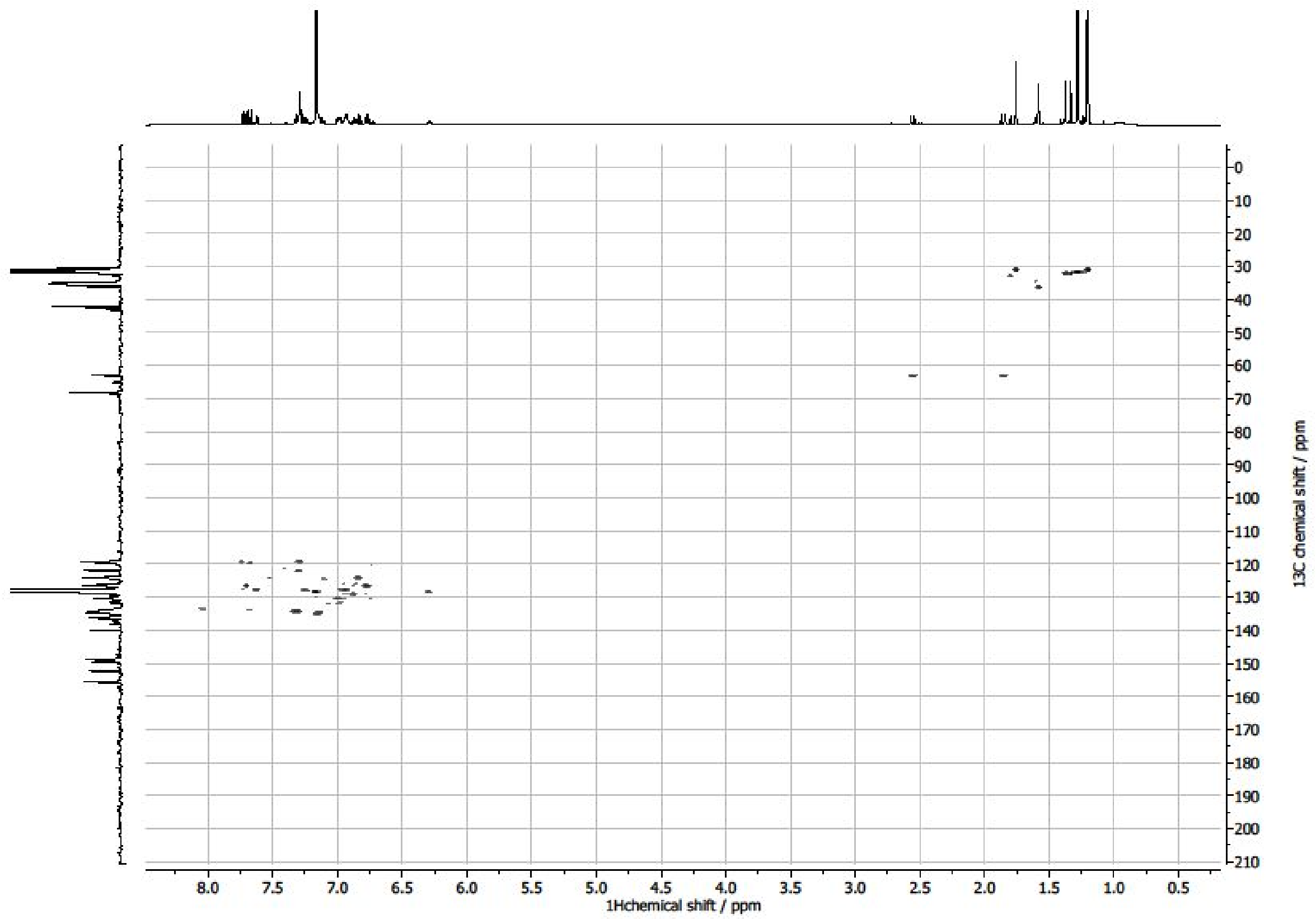

Figure S25. ^{1}H-^{13}C HSQC spectrum (C_6D_6, 298 K) of **4**.

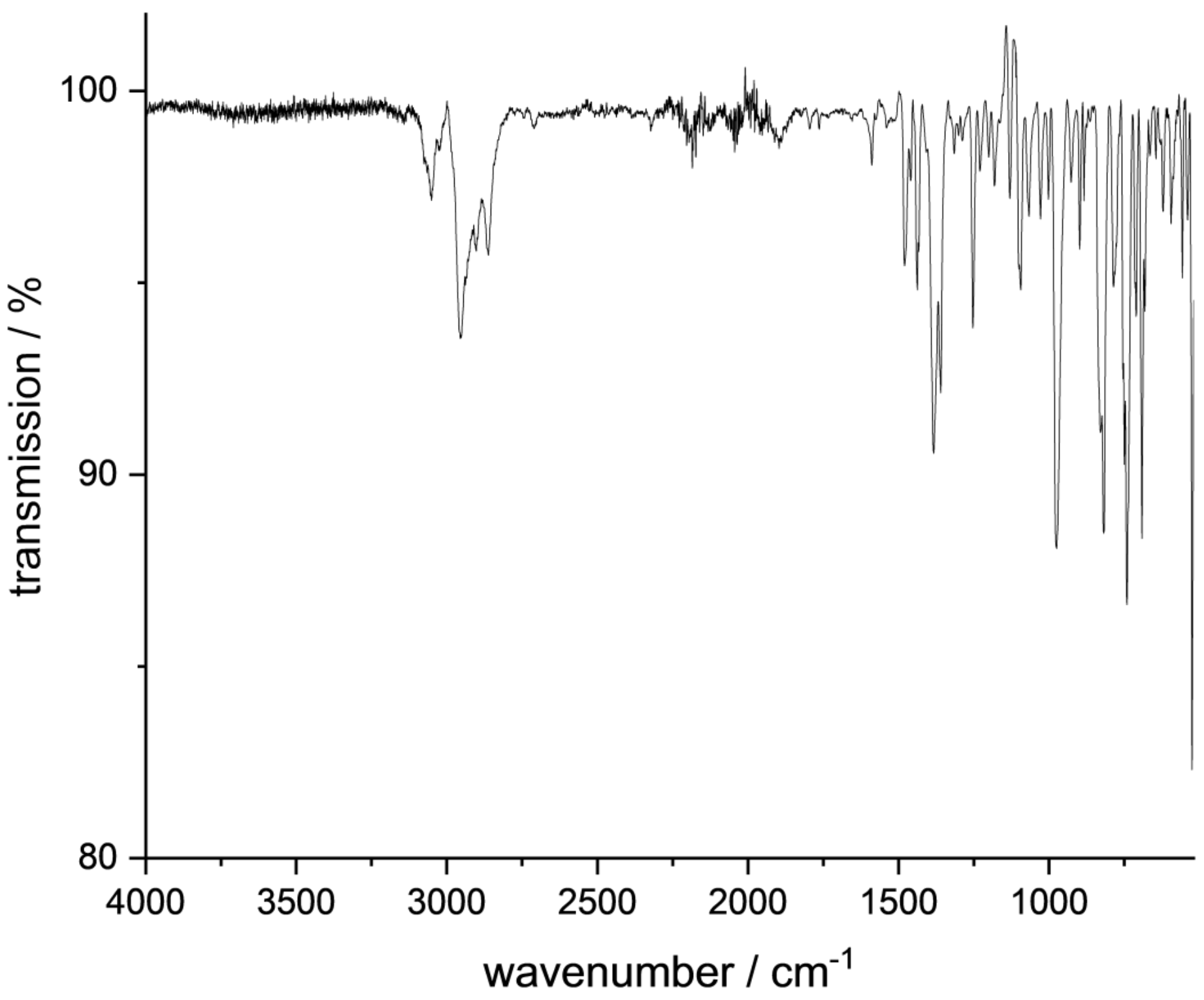

Figure S26. ATR-IR (diamond, cm^{-1}) spectrum of **4**.

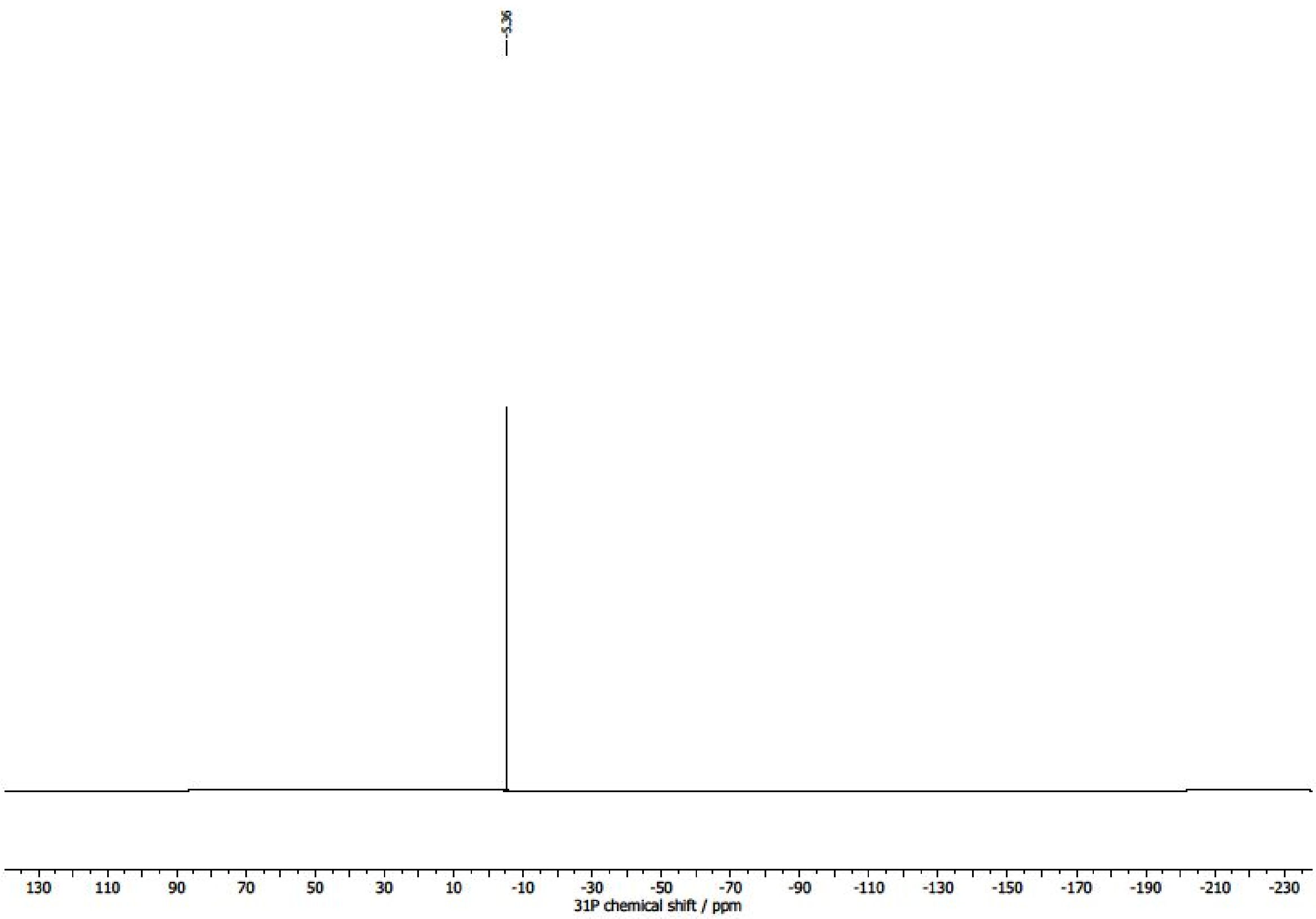

Figure S27. $^{31}P\{^{1}H\}$ NMR spectrum (203 MHz, C_6D_6, 298 K) of triphenylphosphine.

4. Additional computational data

In addition to the computational model used in the main article, a simplified model in which the Fluind rest was replaced by a phenyl ring, was computed as well at the same level of theory, i.e., ωB97X-D3BJ/def2-TZVP//BP86-D3BJ/def2-TZVP.[13]

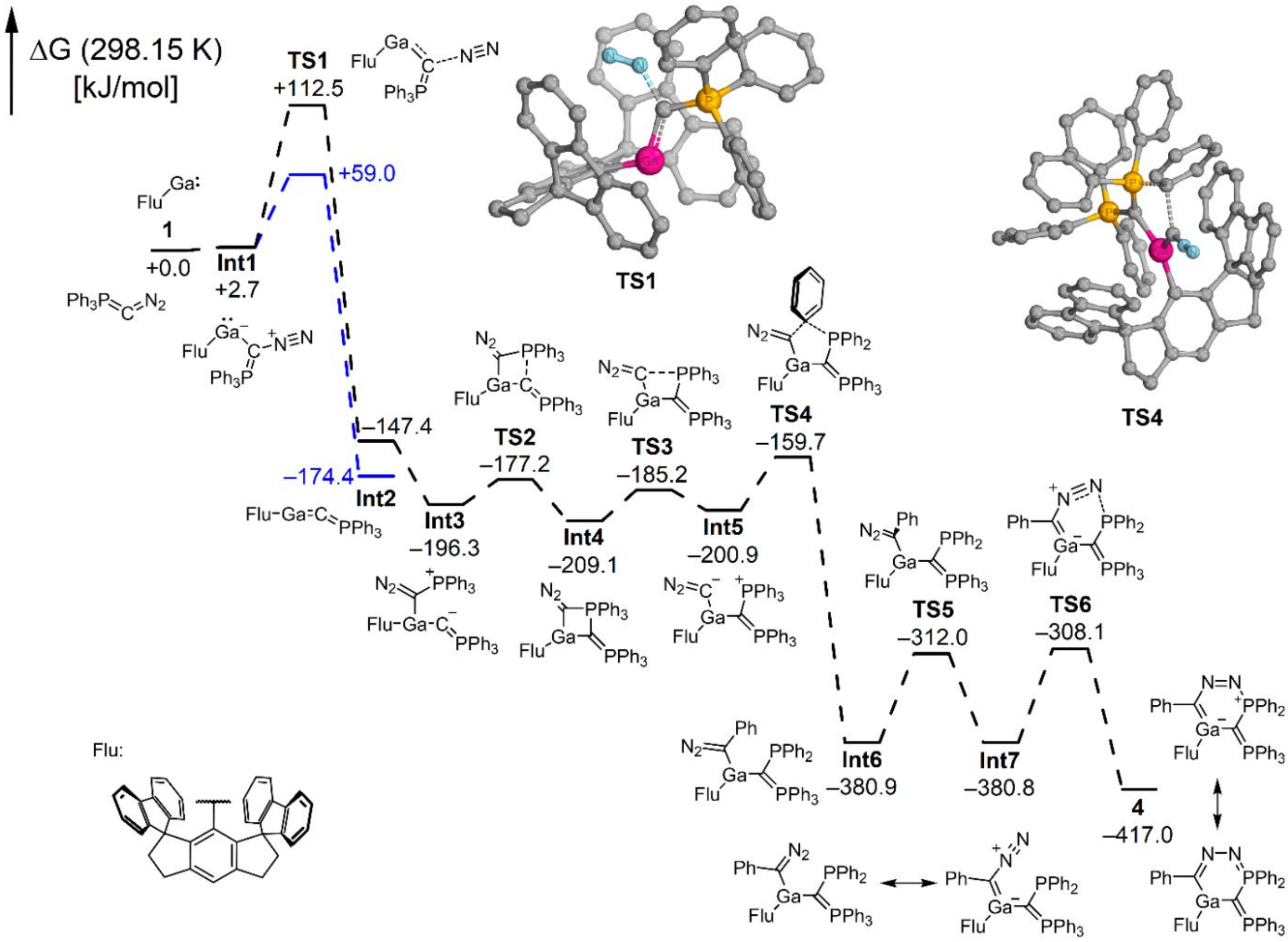

Figure S28. Schematic potential-energy surface with Gibbs free energies at the ωB97X-D3BJ/def2-TZVP//BP86-D3BJ/def2-TZVP level of theory for a simplified model system, i.e., possessing a phenyl ring in place of the Fluind. In addition, **Int1'**, **TS1'** as well as **Int2'** have been modelled at the DLPNO-CCSD(T)/def2-TZVP//BP86-D3BJ/def2-TZVP level of theory (blue line).

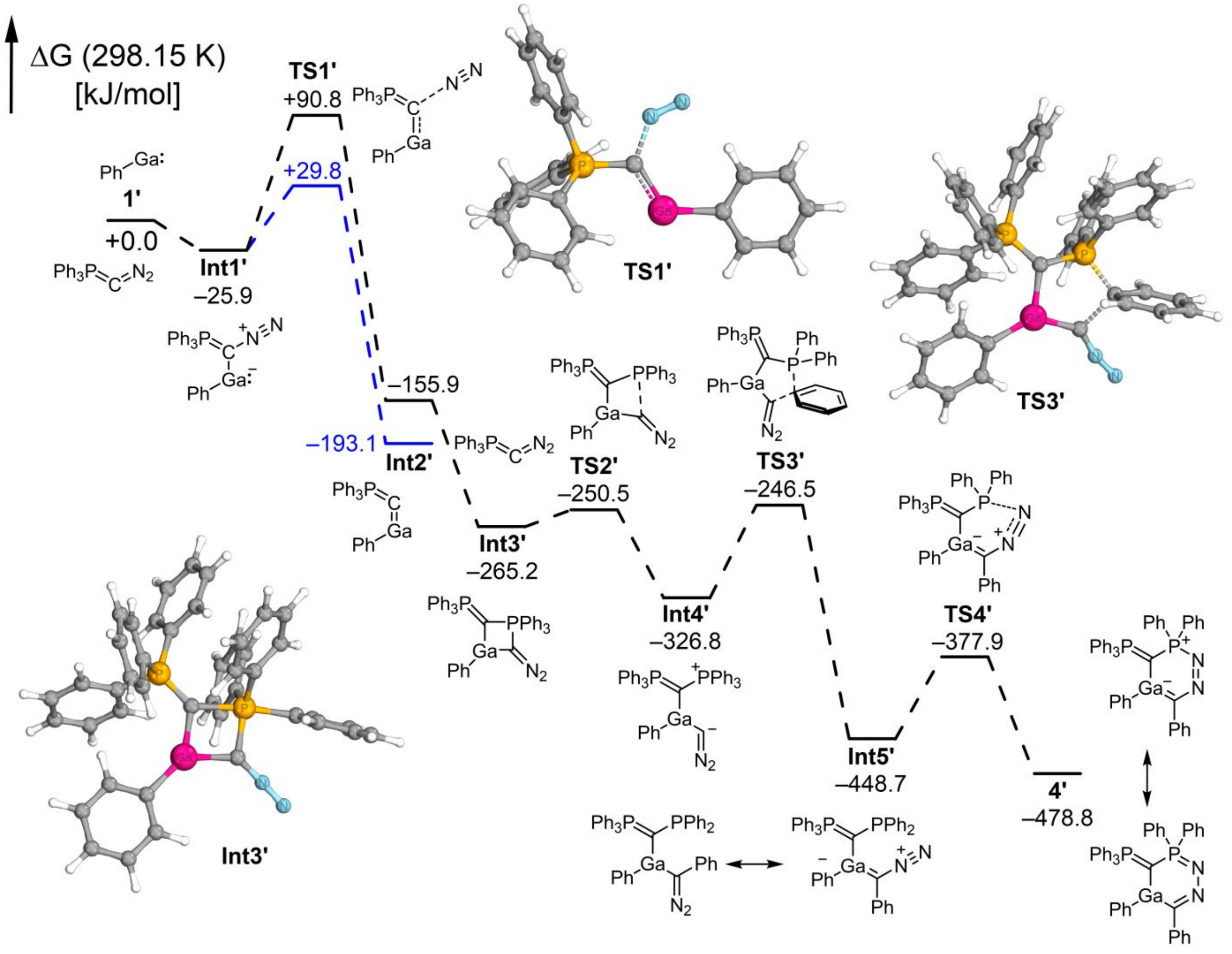

Figure S29. Schematic potential-energy surface with Gibbs free energies at the ωB97X-D3BJ/def2-TZVP//BP86-D3BJ/def2-TZVP level of theory for a simplified model system, i.e., possessing a phenyl ring in place of the Fluind. In addition, **Int1'**, **TS1'** as well as **Int2'** have been modelled at the DLPNO-CCSD(T)/def2-TZVP//BP86-D3BJ/def2-TZVP level of theory (blue line).

5. References

(1) Einstein, A. Über die von der molekularkinetischen Theorie der Wärme geforderte Bewegung von in ruhenden Flüssigkeiten suspendierten Teilchen. *Ann. Phys.* **1905**, *322*, 549–560.

(2) a) Evans, R.; Deng, Z.; Rogerson, A. K.; McLachlan, A. S.; Richards, J. J.; Nilsson, M.; Morris, G. A. Quantitative interpretation of diffusion-ordered NMR spectra: can we rationalize small molecule diffusion coefficients? *Angew. Chem. Int. Ed.* **2013**, *52*, 3199–3202; b) Evans, R.; Dal Poggetto, G.; Nilsson, M.; Morris, G. A. Improving the Interpretation of Small Molecule Diffusion Coefficients. *Anal. Chem.* **2018**, *90*, 3987–3994;

(3) Hiller, W.; Grabe, B.; Schonert, J. Molar Mass Determination for Small and Large Molecules Using Diffusion-Ordered Spectroscopy. *Anal. Chem.* **2024**, *96*, 14902–14908.

(4) Bachmann, S.; Neufeld, R.; Dzemski, M.; Stalke, D. New External Calibration Curves (ECCs) for the Estimation of Molecular Weights in Various Common NMR Solvents. *Chem. Eur. J.* **2016**, *22*, 8462–8465.

(5) Neufeld, R.; Stalke, D. Accurate molecular weight determination of small molecules via DOSY-NMR by using external calibration curves with normalized diffusion coefficients. *Chem. Sci.* **2015**, *6*, 3354–3364.

(6) Tonner, R.; Frenking, G. Divalent carbon(0) chemistry, part 2: Protonation and complexes with main group and transition metal Lewis acids. *Chem. Eur. J.* **2008**, *14*, 3273–3289.

(7) Sheldrick, G. M. SHELXT - integrated space-group and crystal-structure determination. *Acta Cryst. A* **2015**, *71*, 3–8.

(8) Sheldrick, G. M. Crystal structure refinement with SHELXL. *Acta Cryst. C* **2015**, *71*, 3–8.

(9) Hübschle, C. B.; Sheldrick, G. M.; Dittrich, B. ShelXle: A Qt graphical user interface for SHELXL. *J. Appl. Crystallogr.* **2011**, *44*, 1281–1284.

(10) Kratzert, D.; Holstein, J. J.; Krossing, I. DSR: Enhanced modelling and refinement of disordered structures with SHELXL. *J. Appl. Crystallogr.* **2015**, *48*, 933–938.

(11) Spek, A. L. PLATON SQUEEZE: A tool for the calculation of the disordered solvent contribution to the calculated structure factors. *Acta Cryst. C* **2015**, *71*, 9–18.

(12) Dolomanov, O. V.; Bourhis, L. J.; Gildea, R. J.; Howard, J. A. K.; Puschmann, H. OLEX2 : a complete structure solution, refinement and analysis program. *J. Appl. Crystallogr.* **2009**, *42*, 339–341.

(13) a) Perdew, J. P. Density-Functional Approximation for the Correlation-Energy of the Inhomogeneous Electron-Gas. *Phys. Rev. B* **1986**, *33*, 8822–8824; b) Becke, A. D. Density-functional exchange-energy approximation with correct asymptotic behavior. *Phys. Rev. A* **1988**, *38*, 3098–3100; c) Becke, A. D.; Johnson, E. R. A density-functional model of the dispersion interaction. *J. Chem. Phys.* **2005**, *123*, 154101; d)

Weigend, F.; Ahlrichs, R. Balanced basis sets of split valence, triple zeta valence and quadruple zeta valence quality for H to Rn: Design and assessment of accuracy. *Phys. Chem. Chem. Phys,* **2005**, *7*, 3297–3305; e) Johnson, E. R.; Becke, A. D. A post-Hartree-Fock model of intermolecular interactions. *J. Chem. Phys.* **2005**, *123*, 24101; f) Grimme, S.; Antony, J.; Ehrlich, S.; Krieg, H. A consistent and accurate ab initio parametrization of density functional dispersion correction (DFT-D) for the 94 elements H-Pu. *J. Chem. Phys.* **2010**, *132*, 154104; g) Grimme, S.; Ehrlich, S.; Goerigk, L. Effect of the damping function in dispersion corrected density functional theory. *J. Comput. Chem.* **2011**, *32*, 1456–1465; h) Najibi, A.; Goerigk, L. The Nonlocal Kernel in van der Waals Density Functionals as an Additive Correction: An Extensive Analysis with Special Emphasis on the B97M-V and ωB97M-V Approaches. *J. Chem. Theory Comput.* **2018**, *14*, 5725–5738;
